# Effectiveness of Non-Pharmacological Interventions for Reducing Self-Stigma in Adults with Severe Mental Illness: A Systematic Review and Meta-Analysis

**DOI:** 10.3390/healthcare14131841

**Published:** 2026-06-24

**Authors:** Juan Simon Suñer-Adrover, Francisco Vicens-Blanes, Jesús Molina-Mula

**Affiliations:** 1 Psiquiatria, Hospital General Mateu Orfila, 07703 Mahon, Spain; 2 Department of Nursing and Physiotherapy, Universitat de Les Illes Balears, 07122 Palma, Spain; f.vicens@uib.es (F.V.-B.); jesus.molina@uib.es (J.M.-M.)

**Keywords:** severe mental illness, social stigma, hygiene, cosmetics, clothing, stigma reduction interventions

## Abstract

**Aim**: Self-stigma represents a major barrier to recovery among individuals with severe mental illness (SMI). This review aimed to identify and synthesize the available evidence on the effectiveness of non-pharmacological interventions for reducing self-stigma in adults with SMI, while also exploring physical appearance care as a potentially relevant but under-researched area. **Methods**: A systematic review and meta-analysis were conducted in accordance with PRISMA guidelines and the Cochrane Handbook recommendations. The review protocol was registered in PROSPERO (CRD420251013333). Data **Sources**: A comprehensive search was conducted across multiple databases, including the Virtual Health Library, PubMed, Web of Science, the Cochrane Library, and EBSCOhost databases. A snowball search of reference lists was also performed. Studies published in English or Spanish within the past ten years were included. **Review Methods**: Two independent reviewers screened titles, abstracts, and full texts according to predefined criteria. Methodological quality was assessed using the Critical Appraisal Skills Programme España (CASPe). A qualitative synthesis was conducted for all included studies, and a random-effects meta-analysis was performed for studies providing sufficient quantitative data. Standardized mean differences and heterogeneity statistics were calculated. **Results**: Twenty-eight studies were included in the qualitative synthesis, and twelve were eligible for meta-analysis. Multicomponent interventions integrating psychoeducation, cognitive restructuring, narrative approaches, and social support showed the most consistent effects across the evaluated outcome domains. Meta-analytic findings indicated small-to-moderate reductions in self-stigma and improvements in hope, with low levels of statistical heterogeneity across outcomes. Effects on self-esteem, quality of life, self-efficacy, and psychiatric symptomatology were limited or inconsistent across studies. No studies specifically evaluated interventions focused on physical appearance care. **Conclusions**: Non-pharmacological interventions appear to produce modest but potentially meaningful reductions in self-stigma among individuals with SMI, particularly when delivered through multicomponent psychosocial approaches that integrate psychoeducation, cognitive restructuring, narrative techniques, and social support.

## 1. Introduction

Severe mental illness (SMI) is characterized by persistent psychiatric symptoms that markedly impair psychological, social, and emotional functioning [1]. Core psychotic manifestations, including hallucinations, delusions, and disorganized thinking, not only compromise an individual’s ability to live independently but are also associated with a life expectancy that is one to two decades shorter than that of the general population [2]. In addition, SMI is frequently accompanied by mood disturbances, low self-esteem, social anxiety, and broader functional impairments, both of which further diminish quality of life [3].

According to the DSM-5 [4] and the ICD-11 [5], the diagnosis of SMI is based on symptom duration, severity, and the degree of functional impairment in daily life. The complexity of the diagnostic process derives not only from interindividual variability in symptom presentation and the frequent coexistence of psychiatric comorbidities, but also from the elevated burden of preventable physical health conditions observed in this population [6,7]. This functional decline is also associated with a higher prevalence of maladaptive self-care behaviors, including tobacco use, poor diet, and severe physical inactivity [8].

The care of individuals with SMI poses a substantial challenge to public health systems, as it requires comprehensive strategies that address both clinical and social needs [9]. Standard approaches include psychotropic medication, psychological therapies, psychosocial rehabilitation, and community-based support, implemented through diverse national and international policy frameworks [2]. Beyond symptom management, attention to fundamental social determinants such as housing, employment, and interpersonal relationships is essential to promote sustained recovery [10].

In recent decades, increasing attention has been devoted to identifying factors that facilitate both clinical and subjective recovery among individuals with SMI [11].

Within this context, stigma has emerged as a critical barrier to comprehensive recovery. In his seminal work Stigma, Goffman conceptualized stigma as a socially constructed phenomenon arising when particular attributes lead an individual to be perceived as less legitimate or valued within society. Rather than being an inherent characteristic of the individual, stigma is socially constructed through interpersonal interactions, influencing both self-identity and interpersonal relationships [12].

In the field of mental health, stigma encompasses the negative attitudes and beliefs that society applies to individuals with psychiatric diagnoses, often resulting in discrimination, devaluation, and social exclusion [13]. Corrigan and Watson distinguish between public stigma, defined as societal endorsement of prejudicial views, and self-stigma, which occurs when individuals internalize these negative beliefs [14]. The internalization of stigma undermines personal development, restricts academic and occupational advancement, discourages help-seeking, and ultimately erodes self-esteem and quality of life [15].

Evidence consistently demonstrates that individuals with SMI constitute one of the most socially stigmatized groups [16]. They are frequently perceived as aggressive, unpredictable, socially deviant, or incapable of productive participation [17].

Consequently, beyond managing their primary condition, affected individuals must also cope with stigma as an additional psychosocial burden. Indeed, some authors have described stigma as a “second illness” that further constrains opportunities for social inclusion and meaningful life engagement [18].

Self-stigma has therefore become a central construct in contemporary recovery-oriented research [19]. It is understood as the process by which individuals with mental illness internalize societal stereotypes and apply them to themselves, adopting negative self-evaluations that compromise identity and agency [20].

High levels of self-stigma have been associated with symptom exacerbation [21], impaired social functioning [22], reduced self-esteem [20], diminished self-efficacy [21], and lowered expectations of recovery [23]. Moreover, individuals with elevated self-stigma are more likely to disengage from treatment, avoid mental health services, and exhibit poorer adherence, thereby entering a cycle of progressive deterioration [24].

Over the past decade, self-stigma has increasingly been recognized as a systemic challenge for public health systems [25]. This recognition has prompted awareness campaigns [26], legislative initiatives, and broader social efforts aimed at reducing discrimination and facilitating social integration for individuals with SMI [27].

At the clinical level, numerous interventions have been developed to address self-stigma. A systematic review published in 2016 identified several principal approaches, including targeted cognitive behavioral therapy, Narrative Enhancement and Cognitive Therapy, combined cognitive behavioral and social skills training programs, psychoeducational interventions, sociodrama, recovery groups focused on self-image, and peer-led initiatives such as Coming Out Proud and Photovoice [28].

Beyond these established strategies, emerging perspectives have suggested that improvements in physical appearance may influence both self-perception and social perception, thereby potentially contributing to the reduction in stigmatizing attitudes and the promotion of social acceptance. This hypothesis is conceptually linked to the halo effect, a cognitive bias described by Thorndike, whereby the perception of a positive characteristic—such as physical attractiveness—shapes the evaluation of other personal attributes, including competence or trustworthiness. In the context of mental health, improvements in physical appearance have been proposed as a factor that may influence both self-stigma and public stigma; however, empirical evidence supporting this potential relationship remains limited [29].

Given the central role of nurses within multidisciplinary mental health teams, psychiatric nursing professionals may be well positioned to contribute to the implementation of psychosocial interventions aimed at reducing self-stigma [3]. However, empirical evidence regarding the effectiveness of such interventions remains limited [3,28].

In particular, the role of physical appearance care as a potential component of stigma-reduction strategies has received little attention in the literature. This gap became evident during the review process, as no studies specifically evaluating this type of intervention were identified.

Accordingly, the present systematic review aimed to identify and synthesize the available evidence on non-pharmacological interventions that have demonstrated effectiveness in reducing self-stigma among individuals with severe mental illness, while also exploring physical appearance care as a potentially relevant but under-researched factor in stigma reduction.

## 2. Materials and Methods

### 2.1. Design

A systematic review and meta-analysis were conducted to examine non-pharmacological interventions aimed at reducing self-stigma among individuals with severe mental illness, with an exploratory consideration of physical appearance care.

This systematic review included studies with diverse methodological designs, including randomized controlled trials, quasi-experimental studies, observational studies, and descriptive designs. A qualitative synthesis was conducted to integrate findings across all included studies. In addition, a quantitative synthesis (meta-analysis) was performed using only those studies that provided sufficient and comparable statistical data, primarily experimental designs. The study adhered to the PRISMA guidelines for systematic reviews and meta-analyses (see File S1) and to the recommendations of the Cochrane Handbook [30]. The review protocol was registered in the PROSPERO database (CRD420251013333) on 18 March 2025. No significant deviations from the registered protocol were made during the conduct of the review.

### 2.2. Search Strategy

A comprehensive literature search was conducted in May 2025 across the following databases: Virtual Health Library, PubMed, Web of Science, and Cochrane Library. Through the EBSCOhost platform, the following databases were searched: Psychology and Behavioral Sciences Collection, APA PsycInfo, CINAHL with Full Text, Educational Administration Abstracts, MLA Directory of Periodicals, MLA International Bibliography, APA PsycArticles, E-Journals, eBook Collection (EBSCOhost), Social Work Abstracts, and SocINDEX with Full Text.

The search strategy was developed using DeCS and MeSH descriptors combined with Boolean operators. An example of the core search strategy is presented below: (“Mental Disorders” OR “Severe Mental Disorder”) AND (“Social Stigma” OR “Hygiene” OR “Cosmetics” OR “Clothing” OR “Stigma Reduction Intervention”). The search syntax was adapted for each database according to its specific requirements, incorporating Boolean operators (AND, OR), truncation symbols, and database-specific filters.

Complete electronic search strategies are provided in Supplementary File S1, including full search strings, applied filters, truncation strategies, language restrictions, and final search dates, in accordance with PRISMA 2020 recommendations. This Supplementary Material enhances the transparency and reproducibility of the search process. Only studies published in English or Spanish within the past ten years were considered eligible. This temporal restriction was applied to ensure the inclusion of evidence reflecting the most recent developments in psychosocial and recovery-oriented interventions for self-stigma reduction. Given the rapid evolution of therapeutic approaches in mental health, particularly in areas such as cognitive behavioral, narrative, and mindfulness-based interventions, this criterion was intended to enhance clinical relevance, methodological consistency, and applicability of the findings.

The search strategy was primarily structured around the Population (P) and Intervention (I) components of the PICO framework. Outcome (O) and Comparison (C) elements were not explicitly included in the search strings to maximize sensitivity and avoid the omission of potentially relevant studies. This decision was based on the substantial heterogeneity in outcome definitions and measurement instruments related to self-stigma, as well as variability in comparator conditions across psychosocial intervention studies. Outcome and comparison criteria were instead applied during the study selection and eligibility assessment phases.

A complementary snowball search was conducted by screening the reference lists of included studies to identify potentially relevant publications not retrieved through the electronic database search. Study selection proceeded in stages: titles and abstracts were screened first, followed by full-text review to determine eligibility and methodological adequacy. Citation tracking was also performed to identify additional relevant studies. All stages of study selection (title/abstract screening and full-text review) were conducted independently by two reviewers, in accordance with PRISMA 2020 recommendations.

The PRISMA 2020 checklist and complete electronic search strategies are provided in the Supplementary Materials.

### 2.3. Inclusion Criteria

Studies were included if they met the following criteria:

1. Participants were adults (≥18 years) diagnosed with severe mental illness by a psychiatrist.
2. Participants met diagnostic criteria for severe mental illness according to the Dsm-5 [4] or Icd-11 [5].
3. Self-stigma was the primary outcome of interest. Studies were included if self-stigma was assessed as either a primary or secondary outcome to ensure comprehensive identification of relevant evidence. Secondary outcomes included variables such as self-esteem, anxiety, mood, quality of life, self-efficacy, and psychiatric symptomatology.
4. The study reported factors, conditions, or interventions associated with reduced levels of self-stigma in individuals with SMI.
5. The study evaluated a non-pharmacological intervention aimed at reducing self-stigma. Interventions related to physical appearance care were also considered when available.
6. Outcomes included changes in self-stigma and related variables such as self-esteem, anxiety, mood, quality of life, self-efficacy, or psychiatric symptomatology.

### 2.4. Exclusion Criteria

Studies were excluded if:

1. They focused exclusively on forms of stigma other than self-stigma.
2. The intervention was primarily pharmacological and did not include a non-pharmacological component.
3. The publication type corresponded to letters, study protocols, editorials, or expert opinion articles.

### 2.5. Data Collection

The selection process followed four phases: identification, screening, eligibility, and inclusion. Two independent reviewers (JSSA and FVB) conducted the screening process, and discrepancies were resolved through consultation with a third reviewer (JMM).

During the identification phase, duplicate records were removed. In the screening phase, titles were evaluated to exclude clearly irrelevant studies. Potentially eligible articles proceeded to abstract review. Full-text assessment was subsequently conducted to confirm eligibility according to predefined criteria.

Data from included studies were extracted using a structured Excel coding sheet. Extracted variables included study title, authors, year of completion and publication, country and continent of origin, language, study design, data collection methods, setting, recruitment procedures, participant characteristics, risk of bias, analytical approach, and key thematic domains.

In cases of missing or incompletely reported data, efforts were made to contact the study authors to obtain additional information. When such data could not be retrieved, studies were included in the qualitative synthesis if they met the eligibility criteria but were excluded from the meta-analysis when the required quantitative data were insufficient to calculate effect sizes. Missing descriptive information, such as the date of data collection, was reported as not specified and did not affect study inclusion.

Methodological quality was assessed using the Critical Appraisal Skills Programme España (CASPe) checklists, selecting the appropriate tool according to each study design (e.g., randomized controlled trials, cohort studies, and qualitative studies).

Quality assessment was conducted independently by two reviewers (JSSA and FVB), and discrepancies were resolved through discussion with a third reviewer (JMM) until consensus was achieved. Each study was classified as low, moderate, or high methodological quality based on the number of CASPe criteria fulfilled. Studies meeting most of the criteria with minimal risk of bias were classified as high quality, those meeting a moderate number of criteria were categorized as moderate quality, and those with substantial methodological limitations were considered low quality.

Only studies meeting more than six CASPe criteria were retained for analysis to ensure adequate methodological quality, internal validity, and applicability.

Risk of bias in randomized studies included in the meta-analysis was assessed using the Cochrane Risk of Bias 2 (RoB 2) tool [31]. This complementary assessment provided a more detailed evaluation of internal validity in randomized studies, particularly in relation to key domains such as allocation concealment, blinding, and selective reporting.

Levels of evidence were classified according to the Scottish Intercollegiate Guidelines Network (SIGN) framework.

### 2.6. Analysis and Synthesis

#### 2.6.1. Qualitative Synthesis

A qualitative synthesis was undertaken to contextualize the relationship between self-stigma and variables influenced by psychosocial interventions. Six principal domains were identified across the included studies: self-stigma (primary construct), self-esteem, hope, quality of life, psychiatric symptomatology, and self-efficacy.

Secondary outcome domains were defined as follows: self-esteem as an individual’s overall sense of self-worth, hope as a goal-directed motivational state, self-efficacy as an individual’s belief in their ability to perform actions and achieve desired outcomes, quality of life as subjective well-being and functional status, and psychiatric symptomatology as the severity of clinical symptoms. Variability in measurement instruments across studies should be considered when interpreting these outcomes.

The qualitative synthesis was conducted using a structured narrative approach. Studies were systematically organized according to predefined conceptual domains, including self-stigma, self-esteem, quality of life, self-efficacy, psychiatric symptomatology, and hope. Within each domain, intervention characteristics and key findings were compared and synthesized in order to identify patterns, consistencies, and divergences across studies. This approach enabled the integration of evidence from studies with heterogeneous designs while ensuring methodological transparency and analytical coherence.

Studies lacking inferential statistical analyses or not reporting quantitative associations were included exclusively in the qualitative synthesis. Exclusion from the meta-analysis was primarily due to methodological heterogeneity, insufficient comparable data, or non-analytical study designs.

In this context, although the primary objective of this review was to evaluate the effectiveness of non-pharmacological interventions, studies with observational and descriptive designs were also included in the qualitative synthesis. These studies contributed to a broader understanding of the contextual, psychosocial, and structural factors associated with self-stigma, as well as to the identification of relevant outcome domains and underlying mechanisms.

However, only experimental and quasi-experimental studies providing sufficient quantitative data were included in the meta-analysis. This approach allowed for a comprehensive synthesis of the evidence while maintaining methodological rigor in the quantitative analyses.

#### 2.6.2. Quantitative Synthesis

Analytical studies evaluating non-pharmacological stigma-reduction interventions and providing sufficient quantitative data were considered for inclusion in the meta-analysis. The meta-analysis was performed using Meta-Essentials (Version 1.5), an Excel-based meta-analysis workbook developed by Henk van Rhee, Robert Suurmond, and Tony Hak at the Erasmus Research Institute of Management (ERIM), Erasmus University Rotterdam, Rotterdam, The Netherlands. The software was used for all statistical computations.

Outcomes were grouped into six constructs: self-stigma, self-esteem, self-efficacy, quality of life, psychiatric symptomatology, and hope. Self-stigma was considered the primary outcome for both qualitative and quantitative synthesis, while all other constructs (e.g., self-esteem, quality of life, self-efficacy, psychiatric symptoms, and hope) were analyzed as secondary outcomes. Studies assessing multiple outcomes were categorized under each relevant construct.

For each study, pre- and post-intervention means and standard deviations were extracted. Effect size calculations were based on post-intervention means and standard deviations, using the available data reported in each study, and comparisons were conducted between intervention and control groups when available. Change scores were not used due to inconsistent reporting across studies and insufficient data to calculate standardized change effects reliably.

Standardized mean differences were calculated using Hedges’ g correction to account for small sample bias, and effect sizes were weighted using the inverse variance method.

In cases where standard deviations were not directly reported, attempts were made to derive them from available statistical information or to contact study authors. When this was not possible, studies were excluded from quantitative synthesis.

Given the heterogeneity in measurement instruments across studies for similar outcome domains, standardized mean differences were used to enable comparability and pooling of results. This approach allowed effect sizes derived from different scales to be expressed on a common metric.

Publication bias was assessed through visual inspection of funnel plots and Egger’s regression test. The corresponding results are provided in Supplementary File S2. Although no clear evidence of publication bias or small-study effects was observed, these findings should be interpreted cautiously because the limited number of studies included in each meta-analysis may reduce the sensitivity and reliability of these assessment methods.

Formal sensitivity analyses were limited by the small number of studies available for most outcomes. Nevertheless, complementary analyses, including a leave-one-out analysis for the self-stigma construct, were conducted and are reported in Supplementary File S3. Potential sources of bias and heterogeneity were also considered qualitatively in the interpretation of the findings.

A random-effects model was applied due to anticipated clinical and methodological heterogeneity. Baseline equivalence was examined by comparing pre-test means between groups.

Heterogeneity was assessed using Cochran’s Q test and the I^2^ statistic with corresponding 95% confidence intervals. I^2^ values above 50% were considered indicative of substantial heterogeneity. Effect sizes were interpreted according to Cohen’s criteria (0.2 small, 0.5 moderate, 0.8 large). Statistical significance was set at *p* < 0.05.

## 3. Results

### 3.1. Search Results

The literature search was completed in May 2025 and yielded 8744 records through database searching, in addition to 26 records identified through the snowball technique. After removal of 7255 duplicates, 1489 unique records remained and underwent title and abstract screening.

Following this screening process, 1458 records were excluded. A total of 31 reports from database searches were sought for retrieval, while 26 additional records were identified through snowballing. Of these, 17 could not be retrieved. In total, 40 articles were assessed for full-text eligibility.

**Figure 1:**
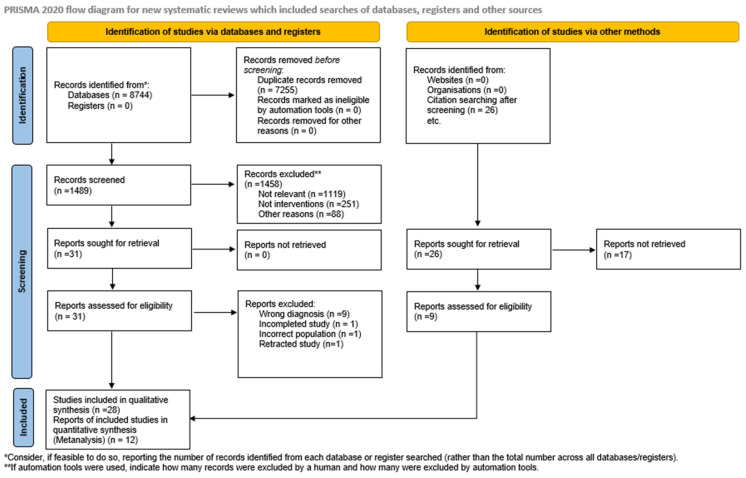
PRISMA 2020 flow diagram of the study selection process.

Of these, 12 studies were excluded for failing to meet the predefined inclusion criteria. The reasons for exclusion included non-compliance with diagnostic criteria (n = 9), incorrect population (n = 1), Incomplete reporting (n = 1), and article retraction (n = 1). Ultimately, 28 studies were included in the qualitative synthesis, of which 12 were incorporated into the meta-analysis. The study selection process is presented in the PRISMA flow diagram (Figure 1) [32].

The included studies were subsequently categorized and summarized according to their main methodological and clinical characteristics. For each study, information was extracted on the author, study design, data collection method, study objectives, setting and period of data collection, population and sample characteristics, main findings, conclusions, methodological quality assessment, and level of evidence. Methodological quality was critically appraised using the Critical Appraisal Skills Programme España (CASPe) [33], whereas levels of evidence were classified according to the Scottish Intercollegiate Guidelines Network (SIGN) framework [34].

For studies included in the meta-analysis, additional data were extracted, including the type of intervention and comparator, the number of participants allocated to the intervention and control groups, the measurement instruments employed, and the outcome variables analyzed.

A detailed description of all studies included in the qualitative and quantitative synthesis is provided in Supplementary Tables S1 and S2.

### 3.2. Description of Included Studies

The 28 studies included in this review exhibited substantial methodological heterogeneity. Ten randomized experimental studies (35.71%) were identified [35,36,37,38,39,40,41,42,43,44], followed by seven descriptive cross-sectional studies (25%) [45,46,47,48,49,50,51] and six observational cross-sectional studies (21.43%) [52,53,54,55,56,57]. In addition, two descriptive longitudinal studies (7.14%) were included [58,59]. The remaining studies comprised one quasi-experimental study without a control group (3.4%) [60], one experimental study employing a four-group Solomon design (3.57%) [61], and one systematic review (3.57%) [62].

**Figure 2:**
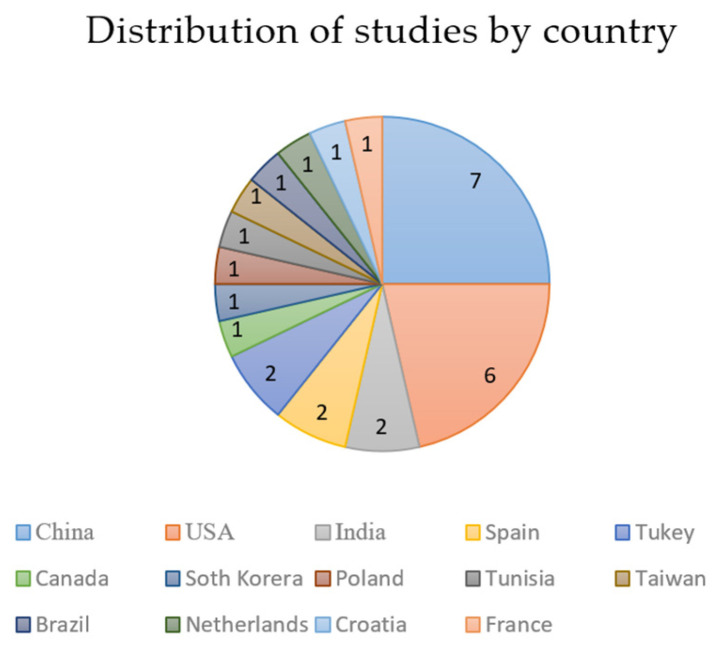
Distribution of studies by country.

The included studies showed broad geographical representation. The largest proportion were conducted in China (n = 7; 24.1%) and the United States (n = 6; 20.7%), followed by India (n = 2; 6.9%), Spain (n = 2; 6.9%), and Turkey (n = 2; 6.9%). One study each (3.4%) was conducted in Canada, South Korea, Poland, Tunisia, Taiwan, Brazil, the Netherlands, Croatia, and France. The geographical distribution of the included studies is illustrated in Figure 2.

Regarding the period of data collection, three studies (10.7%) were conducted within the past five years, 12 studies (42.9%) were carried out in earlier periods, and 13 studies (46.4%) did not specify the date of data collection.

In terms of data collection methods, most studies employed structured questionnaires and validated psychometric scales, administered either as self-report measures or through clinical interviews. In several instances, face-to-face structured interviews were conducted [47,52].

The instruments used to assess self-stigma in the studies included in the meta-analysis were predominantly specific, widely validated scales. Notably, ten of the twelve studies included in the meta-analysis employed the Internalized Stigma of Mental Illness (ISMI) scale, a widely validated instrument designed to assess the extent to which individuals with mental illness internalize stigmatizing societal attitudes. This methodological homogeneity enhances cross-study comparability and strengthens the robustness of pooled estimates.

A smaller number of studies used the Perceived Devaluation–Discrimination Scale (PDD), which focuses on individuals’ perceptions of negative societal attitudes. Elevated PDD scores have been consistently associated with higher levels of internalized stigma [40,41].

Self-esteem was assessed using instruments widely established in mental health research. The most frequently employed measure was the *Rosenberg Self-Esteem Scale* (RSES), which evaluates global self-worth and was used in several studies included in the meta-analysis [35,42,43]. Other studies used the *Self-Esteem Rating Scale* (SERS), which differentiates between positive and negative dimensions of self-esteem [38], as well as a culturally adapted Chinese version of the *Self-Esteem Scale* (SES) [36].

Greater heterogeneity was observed in the measurement of quality of life. Four different instruments were employed: the *Schizophrenia Quality of Life Scale* (SQLS), specific to individuals with schizophrenia and in which higher scores indicate poorer quality of life [36,40], *the Satisfaction With Life Scale* (SWLS), focused on global life satisfaction [43], the *Manchester Short Assessment of Quality of Life* (MANSA), which assesses perceived quality of life across multiple domains [38]; and the *Quality of Life Scale* (QLS), oriented toward functioning and well-being in individuals with schizophrenia [42].

Self-efficacy was evaluated using instruments reflecting partially distinct conceptual frameworks. The *General Self-Efficacy Scale* (GSES), which measures overall perceptions of personal competence, was used in some studies [44]. Other investigations employed more specific measures, such as the *Belief in One’s Own Working Capacity* subscale, focused on work-related self-efficacy [63], or broader instruments such as the *Boston University Empowerment Scale* (BUES), in which self-efficacy constitutes one dimension of a larger empowerment construct [61].

Psychiatric symptomatology was primarily assessed using standardized instruments widely employed in clinical research. The *Positive and Negative Syndrome Scale* (PANSS) was the most frequently used measure to evaluate positive symptoms, negative symptoms, and general psychopathology [38,42]. In addition, some studies utilized the *Brief Psychiatric Rating Scale* (BPRS), which has a structure and evaluative framework comparable to that of the PANSS [36]. A smaller number of studies incorporated the *Beck Depression Inventory–II* (BDI-II), focusing specifically on depressive symptomatology [35].

Finally, hope was assessed using two psychometric instruments capturing complementary aspects of the construct. The Herth Hope Index (HHI), designed to measure general levels of hope, was used in several studies [35,40]. The Work Hope Scale (WHS) assessed hope specifically in relation to vocational domains [63]. While the use of these instruments allows for the assessment of different dimensions of hope, it also introduces conceptual variability that should be considered when interpreting pooled findings.

Table 1 summarizes the scales used to assess each outcome domain across the studies included in the meta-analysis, including measures of self-stigma, self-esteem, quality of life, self-efficacy, psychiatric symptomatology, and hope.

Across the included studies, participants were recruited predominantly from hospital and outpatient settings, including community mental health services, psychiatric hospitals, and rehabilitation units. Some investigations were carried out in rural community contexts [50] or within sheltered employment settings [58], thereby broadening the contextual scope of stigma experiences across diverse social environments.

With respect to sociodemographic characteristics, most studies included mixed samples of adult men and women with severe mental illness, representing approximately 86.2% of the total sample. Two studies exclusively recruited women [40,41], all of which focused on hospitalized women diagnosed with schizophrenia. One study included only male participants [63], targeting men with psychiatric disabilities enrolled in vocational empowerment programs.

Participant ages ranged from 18 to 91 years, with the majority of samples comprising young to middle-aged adults (18–65 years). Some studies included older adults, particularly in rural community settings.

The most frequently represented diagnoses were schizophrenia, bipolar disorder, major depressive disorder, and schizophrenia spectrum disorders. Diagnoses were established according to DSM-IV, DSM-5, or ICD-10 criteria and, in several cases, were confirmed by psychiatrists through structured clinical interviews.

Sample sizes varied substantially across studies, ranging from 51 participants in the study by Russinova et al. [63], which examined vocational empowerment in individuals with psychiatric disabilities, to 1403 participants in the multicenter study by Grover et al. [54], which investigated self-stigma among individuals with severe mental illness in India. Larger samples were typically observed in multicenter descriptive cross-sectional studies, whereas experimental designs generally involved smaller, intervention-specific cohorts.

### 3.3. Variables Measured in Anti-Stigma Interventions

#### 3.3.1. Qualitative Analysis

The qualitative synthesis classified studies according to the primary and secondary variables assessed in anti-stigma interventions. Intervention effectiveness was evaluated based on changes observed in stigma-related scales and their associations with psychosocial, clinical, and functional outcomes.

Self-stigma was assessed in nearly all studies (93.1%) [47,54,59]. Other frequently examined variables included psychiatric symptomatology (37.93%) [38,42], self-esteem (34.48%) [35,51], quality of life (17.24%) [36], self-efficacy (13.79%) [44] and hope (10.34%) [40]. Less frequently assessed variables (below 7%) included coping style [35,39], personal and social functioning [36,59] and vocational identity [63].

A broad range of intervention modalities was identified. These included group-based cognitive–behavioral interventions [43], narrative-based programs [42], expressive writing [40] and psychoeducational approaches [41,60]. Approximately 65% of the studies implemented a single intervention modality, whereas 35% combined multiple therapeutic components, such as psychoeducation, cognitive restructuring, and narrative work [35], vocational empowerment [63] or tools such as photovoice [39]. Interventions were delivered across hospital, community, and outpatient settings and were evaluated using standardized validated instruments.

Overall, multicomponent psychosocial interventions combining psychoeducation, cognitive-behavioral strategies, and narrative or empowerment-based elements emerged as the most consistently effective approach across outcome domains. In contrast, single-component interventions showed more variable effects, with less consistent impact across psychosocial and clinical variables.

For inclusion in the meta-analysis, variables were selected based on both conceptual relevance and frequency of reporting. The six dimensions retained were self-stigma, self-esteem, quality of life, self-efficacy, psychiatric symptomatology, and hope. These variables were selected based on the availability of sufficiently consistent quantitative data across studies, thereby enabling statistical aggregation and comparison.

#### 3.3.2. Quantitative Analysis and Meta-Analysis

The meta-analysis included 12 empirical studies evaluating anti-stigma interventions in individuals with severe mental illness. One study [37] was excluded due to insufficient reporting of outcome data and attempts to obtain additional information from the authors were unsuccessful.

Six outcome variables were analyzed: self-stigma, self-esteem, self-efficacy, quality of life, clinical symptomatology, and hope.

During the analytical process, effect sizes exceeding ±1 were identified in certain comparisons. These effect sizes were examined as potential outliers and were excluded to reduce the influence of extreme observations that could disproportionately affect pooled estimates. This decision was based on the limited number of studies and the need to preserve comparability across analyses. However, this approach may introduce bias and has therefore been considered in the interpretation of results.

Although formal sensitivity analyses were limited by the small number of studies available for most outcomes, robustness was explored through complementary methodological strategies. Outcome variables were grouped into broader conceptual constructs, with one meta-analysis conducted per construct to enhance comparability across studies.

For the self-stigma construct, which included the largest number of studies, a leave-one-out sensitivity analysis was conducted by sequentially removing each study to evaluate the stability of the results. This procedure did not meaningfully alter the pooled estimates or heterogeneity, which remained at I^2^ = 0%, supporting the stability of the findings.

Across all six outcome domains, no statistical heterogeneity was observed (I^2^ = 0%), suggesting limited between-study variability. Although this finding may appear unusual, it should be interpreted with caution. The small number of studies included in each analysis limits the statistical power of heterogeneity tests, which may result in an underestimation of true between-study variability. Additionally, the use of similar outcome measures across studies may have contributed to the observed consistency.

**Figure 3:**
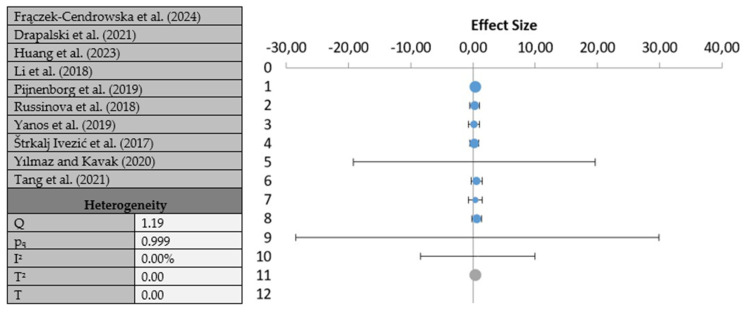
Analyzed data for the self-stigma variable (see Refs. [35,36,38,39,41,42,43,44,60,61]).

**Figure 4:**
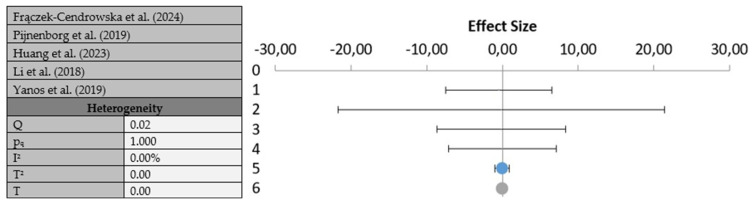
Analyzed data for the self-esteem variable (see Refs. [35,36,38,42,43]).

**Figure 5:**
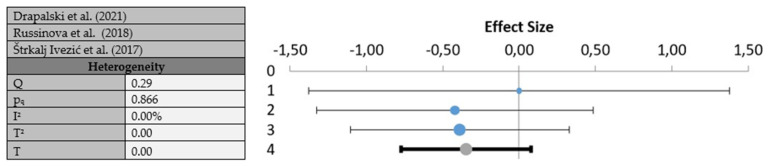
Analyzed data for the self-efficacy variable (see Refs. [39,44,61]).

**Figure 6:**
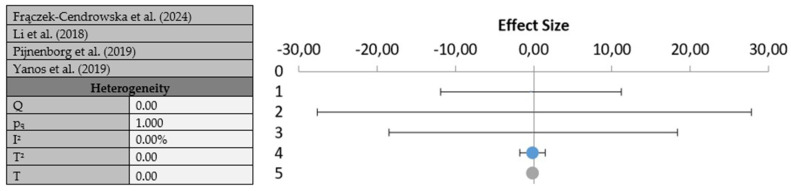
Analyzed data for the quality-of-life variable (see Refs. [36,38,42,43]).

**Figure 7:**
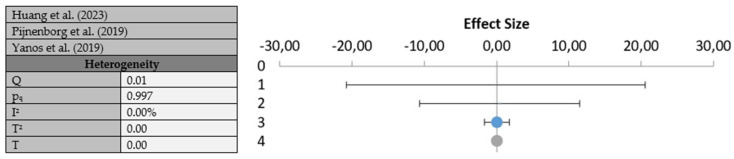
Analyzed data for the clinical symptomatology variable (see Refs. [35,38,42]).

**Figure 8:**
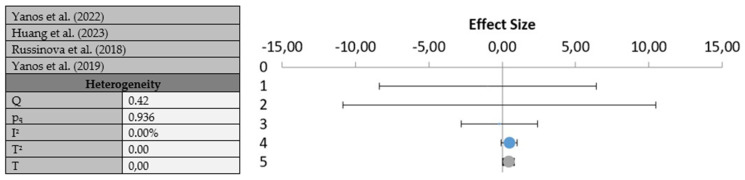
Analyzed data for the hope variable (see Refs. [20,35,39,42]).

Regarding the magnitude of effects, hope demonstrated the largest positive effect size (ES = 0.44), followed by self-stigma (ES = 0.34), both corresponding to small-to-moderate effects according to conventional benchmarks. Self-efficacy showed a negative effect size (ES = −0.35), also within the small-to-moderate range, suggesting a reduction in the outcome as measured across the included studies. Quality of life (ES = −0.18) and self-esteem (ES = −0.05) exhibited smaller negative effects with limited clinical significance, while psychiatric symptomatology showed a negligible positive effect (ES = 0.05). These findings are presented graphically in Figure 3, Figure 4, Figure 5, Figure 6, Figure 7 and Figure 8.

### 3.4. Risk of Bias

Risk of bias was assessed using the Cochrane Risk of Bias 2 (RoB 2) tool [31]. Overall, most studies included in the meta-analysis demonstrated a low risk of bias. Nine studies were classified as low risk [35,36,38,40,41,42,44,63], whereas four were rated as having a moderate risk of bias [37,43,60,61]. No studies were classified as high risk.

At the domain level, concerns were primarily identified in allocation concealment and blinding procedures. Two studies presented limitations in allocation concealment [60,61], and four studies reported deficiencies in blinding [37,43,60,61].

**Figure 9:**
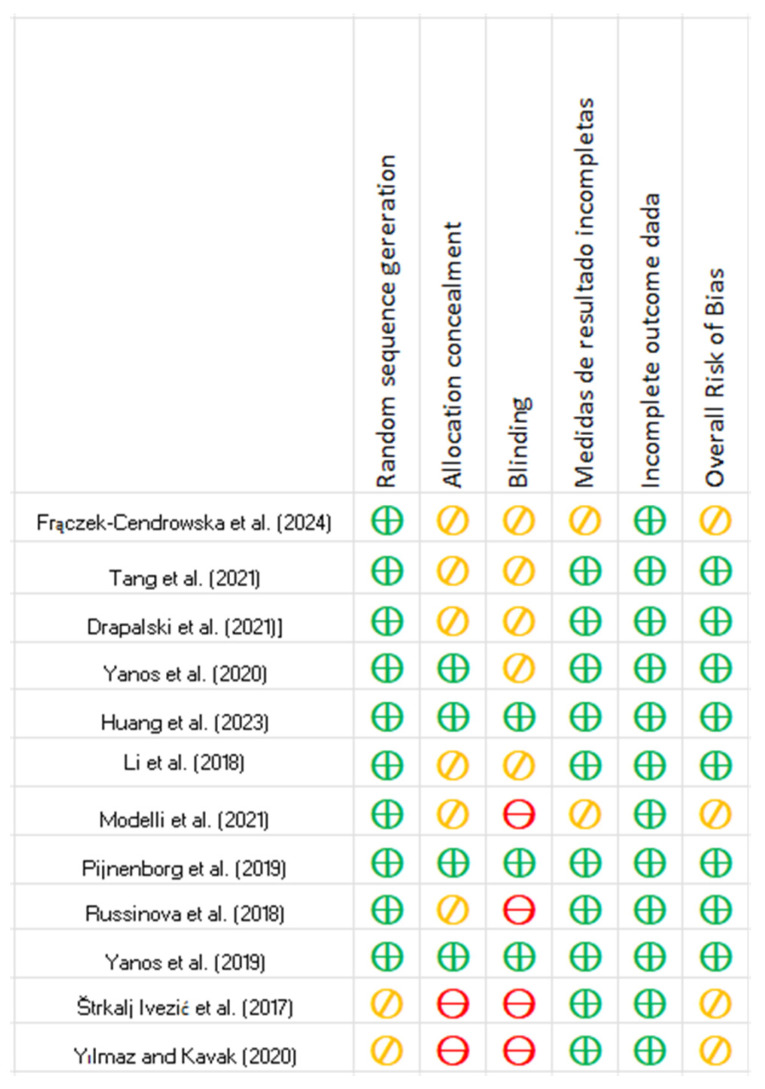
Risk of bias summary according to the RoB 2 tool. Green circles indicate low risk of bias, yellow circles indicate some concerns (moderate risk of bias), and red circles indicate high risk of bias. (see Refs. [20,35,36,37,38,39,41,42,43,44,60,61]).

**Figure 10:**
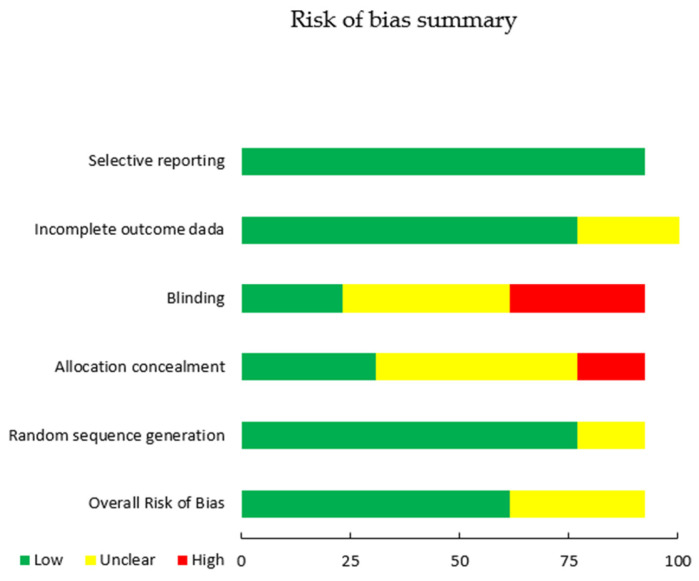
Risk of bias summary. Green indicates low risk of bias, yellow indicates some concerns (moderate risk of bias), and red indicates high risk of bias.

No high risk of bias was detected in domains related to sequence generation, handling of incomplete outcome data, or selective reporting, suggesting generally adequate methodological rigor and transparency in outcome reporting. The detailed risk-of-bias assessments are presented graphically in Figure 9 and Figure 10.

## 4. Discussion

This systematic review and meta-analysis examined the effectiveness of anti-stigma interventions for individuals with severe mental illness (SMI), focusing on key outcome domains including self-esteem, quality of life, self-efficacy, psychiatric symptomatology, and hope, as well as contextual factors influencing these dimensions.

In this review, clinical improvement refers to a multidimensional construct that includes changes in both the primary outcome (self-stigma) and secondary outcomes such as psychiatric symptoms, quality of life, self-esteem, and self-efficacy.

The apparent consistency of effect sizes should be interpreted with caution. Although statistical heterogeneity was not detected, this finding may be influenced by the limited number of studies included in each analysis, as well as similarities in outcome measures and intervention characteristics. Additionally, methodological differences across studies, including variations in design, duration, and implementation, may not be fully captured by statistical indicators alone.

In addition to these considerations, a key limitation of this review is the substantial clinical and methodological heterogeneity across the included studies. The interventions analyzed comprised a wide range of approaches, including psychoeducational programs, cognitive-behavioral therapies, narrative-based interventions, peer-support initiatives, and broader psychosocial and observational studies.

This diversity, while reflecting the complexity of self-stigma as a construct, may limit the interpretability and direct clinical applicability of the pooled quantitative estimates. Differences in intervention content, duration, delivery format, and target populations make it challenging to isolate specific active components and to generalize findings across settings.

Furthermore, subgroup analyses based on intervention type, diagnostic categories, or study design were not performed due to the limited number of studies available within each subgroup, which precluded meaningful statistical comparisons. Future research with larger and more homogeneous samples would be necessary to enable more refined analyses, including subgroup and stratified analyses based on intervention type, diagnosis, and study design, thereby strengthening the clinical applicability of the findings.

In addition, the risk of bias identified across several studies should be considered when interpreting these findings. Although most studies were classified as having a low overall risk of bias, a subset presented moderate risk in domains such as allocation concealment [60,61] and blinding [37,43,60,61]. These methodological limitations may have influenced the observed effect sizes and should be taken into account when assessing the robustness of the pooled estimates.

A formal sensitivity analyses excluding studies with moderate risk of bias was not conducted due to the limited number of studies included in each meta-analysis. However, the potential impact of these biases has been considered qualitatively in the interpretation of results.

Beyond overall effectiveness, an important aspect to consider is the specific characteristics of interventions that may explain variability in outcomes.

Overall, structured psychological interventions demonstrated moderate effectiveness in reducing internalized stigma and strengthening personal resources such as self-esteem, hope, and self-efficacy [41,43,44]. However, findings are not uniform. For example, a narrative-based cognitive therapy program reduced perceived stigma without producing significant improvements in self-esteem, hope, or depressive symptoms [35], whereas Li et al. reported improvements in psychotic symptoms and social functioning without corresponding reductions in self-stigma [36]. Similarly, the HOP intervention yielded only marginal decreases in stigma-related stress [37] and REFLEX improved clinical insight but did not significantly affect self-esteem or depressive symptomatology [38].

By contrast, interventions incorporating positive psychology and narrative components appear to generate broader and more sustained effects. Expressive writing grounded in positive psychology significantly improved hope, coping strategies, and quality of life, while also reducing perceived stigma [40]. These findings suggest that interventions promoting positive self-reflection and meaning-making may contribute to improvements across multiple recovery-related outcomes.

In addition to intervention characteristics, broader contextual and structural influences should also be considered when interpreting these findings.

The findings of this review suggest that contextual factors may play an important role in shaping the experience and reduction in self-stigma. Factors such as social support, socioeconomic conditions, cultural background, and access to mental health services appear to play a significant role in moderating intervention outcomes. In particular, supportive interpersonal environments may facilitate engagement with psychosocial interventions and enhance their effectiveness, while structural barriers such as stigma, unemployment, or limited access to care may hinder recovery processes. These aspects underscore the need to interpret intervention effectiveness within a broader social and contextual framework.

Taken together, multimodal interventions integrating psychoeducation, cognitive restructuring, mindfulness, and narrative approaches tend to yield clinically meaningful improvements. Nevertheless, their impact on self-esteem, hope, and quality of life appears contingent upon baseline characteristics, intensity and specificity of intervention content, and cultural contextualization. These considerations justify a domain-specific analysis.

Despite these general patterns, the impact of interventions appears to vary across outcome domains, highlighting the complexity of their effects on different aspects of recovery.

In terms of self-stigma, the available evidence suggests that multicomponent interventions—particularly those integrating cognitive-behavioral, narrative, and empowerment-based elements—demonstrated the most consistent reductions in internalized stigma. Within this general pattern, several studies reported significant improvements associated with mindfulness-based approaches [60], group psychoeducation [61], and empowerment-oriented components such as the Vocational Empowerment Program (VEP) [63], while other interventions produced more limited or inconsistent effects.

Narrative Enhancement and Cognitive Therapy (NECT) demonstrated comparable reductions in Taiwan [42], although its implementation in the United States yielded more modest effects. Conversely, interventions such as Ending Self-Stigma [44] and REFLEX [38] did not show statistically meaningful changes.

The ISMI subdimensions most responsive to intervention were social withdrawal and stereotype endorsement, particularly in NECT, VEP, and mindfulness-based programs. Improvements in alienation and perceived discrimination were also reported, especially in outpatient NECT samples. Stigma resistance improved significantly in VEP, whereas mindfulness-based interventions did not yield comparable effects [60].

In contrast to self-stigma, no consistent pattern of effectiveness was observed for self-esteem across interventions, with some interventions producing modest or short-term improvements while others failed to demonstrate significant or sustained effects [35,42]. However, other studies reported significant within-group improvements using the Rosenberg Self-Esteem Scale in a brief cognitive-behavioral intervention explicitly targeting self-perceptions [43].

Similarly, another study observed significant improvements at six months in a comprehensive community-based intervention in China, although these effects were not sustained at nine months, suggesting short-term gains without long-term consolidation [36]. The Dutch REFLEX trial, assessed using the Self-Esteem Rating Scale, found no significant changes in either positive or negative subdimensions and no between-group differences, reinforcing the pattern of limited sustained impact [38].

Similarly, no clear pattern of effectiveness emerged for quality of life outcomes, with some interventions—particularly those grounded in positive psychology—demonstrating meaningful improvements [40], while others showed limited or non-specific changes, possibly influenced by non-specific therapeutic factors such as group participation [36].

The Polish cognitive-behavioral program did not produce significant between-group differences [43]. Similarly, REFLEX and NECT showed improvements in both intervention and comparison groups without statistically significant between-group differences [38,42], suggesting the potential influence of non-specific therapeutic factors such as group participation.

Similarly, no consistent pattern of effectiveness was identified for self-efficacy across interventions, with results varying substantially between studies. While the Ending Self-Stigma program did not produce significant post-treatment or follow-up differences [44], evidence regarding self-efficacy outcomes remained limited and heterogeneous across the included studies.

Likewise, the VEP intervention yielded significant improvements in work-related self-efficacy and overall empowerment [63]. However, Ivezić et al. found no significant between-group differences despite reductions in self-stigma, possibly reflecting the program’s emphasis on conceptual understanding rather than active skill acquisition [61].

Conversely, REFLEX and NECT trials did not show meaningful symptomatic improvements despite psychosocial gains [38,42]. The Chinese community-based intervention provided more consistent evidence of symptom reduction at six and nine months [36], whereas depressive symptoms remained stable and within normal ranges in the Taiwanese NECT study [35].

Finally, hope emerged as one of the most responsive constructs across interventions. The positive emotional writing intervention produced statistically significant increases in hope [40]. In contrast, NECT did not significantly modify hope scores among hospitalized patients [35].

The VEP intervention generated sustained increases in work-related hope, suggesting that hope may be domain-specific and particularly sensitive to interventions targeting functional empowerment [63].

Beyond the variability observed across outcome domains, it is also important to consider broader determinants influencing the development and persistence of self-stigma.

Although no studies included in this review directly evaluated interventions specifically focused on physical appearance or appearance-based care, the following considerations should be interpreted as theoretical implications and potential directions for future research rather than conclusions supported by direct empirical evidence.

### Determinants of Self-Stigma

Beyond intervention type, the literature highlights contextual and structural determinants influencing self-stigma. Social support consistently functions as a mediator between stigma and both self-esteem and recovery outcomes [52,64].

Self-stigma also negatively affects therapeutic engagement and recovery trajectories, as internalized stereotypes are associated with reduced treatment adherence, diminished motivation, and limited utilization of community resources [53].

Structural determinants, including access to healthcare, employment opportunities, and inclusive public policies, play a protective role. Employment participation and community integration are consistently associated with improved psychological and functional outcomes [63,64].

Subjective cognitive complaints have also been identified as potential mediators between stigma and self-esteem [51], reinforcing the importance of integrating cognitive assessment and remediation strategies into anti-stigma interventions.

Finally, cultural factors significantly influence the experience of stigma. Studies conducted in Asia and Africa indicate that stigma may be more pronounced in rural settings, among women, and among individuals with lower socioeconomic status [50,54,57]. These findings underscore the necessity of culturally and socioeconomically tailored interventions to maximize relevance and effectiveness.

However, subgroup analyses based on geographical or cultural context were not performed, due to the limited number of studies available within each region, which precluded meaningful statistical comparisons. This represents a limitation of the current meta-analysis and highlights an important area for future research, as larger and more geographically balanced samples would be necessary to enable robust context-specific analyses and more clinically applicable recommendations.

These findings should be interpreted in light of the methodological limitations outlined above, particularly the restriction to studies published after 2015, which may have resulted in the exclusion of earlier foundational research.

## 5. Limitations of the Study

This review is subject to several key limitations that should be considered when interpreting the findings. These include: (1) substantial clinical and methodological heterogeneity across interventions; (2) the inclusion of observational and descriptive studies in the qualitative synthesis; (3) potential language and selection bias due to restrictions to English and Spanish publications and to studies published within the past ten years; (4) the absence of studies directly evaluating appearance-based interventions; and (5) limitations related to the meta-analytic component, including small sample sizes, the limited reliability of sensitivity and publication bias assessments due to the small number of available studies, and variability in outcome measures.

Despite the advances identified across the analyzed domains, several methodological and conceptual limitations should be considered when interpreting and generalizing these findings.

First, not all interventions produced significant improvements in clinical outcomes such as psychiatric symptomatology or affective well-being, suggesting that effectiveness is highly dependent on the nature of the intervention and the settings in which it is implemented.

Second, the meta-analysis was constrained by incomplete numerical reporting in some studies and by the predominance of immediate post-intervention assessments. Long-term follow-up data were limited, restricting the ability to evaluate the durability and sustainability of observed effects.

Furthermore, variability in the measurement instruments complicated direct comparisons across studies. Although variables were grouped into broader conceptual constructs to facilitate synthesis, this approach may obscure nuances associated with specific scales and should therefore be interpreted with caution.

The review was also limited to studies published from 2015 onward and to those written in English and Spanish. While these criteria ensured the inclusion of contemporary and accessible evidence, they may have resulted in the exclusion of earlier or non-English studies. Therefore, potential language and selection biases cannot be ruled out and should be considered when interpreting the findings.

Although formal sensitivity analyses were constrained by the limited number of studies per outcome, complementary approaches, including a leave-one-out analysis for the self-stigma construct, were conducted. However, the robustness of these assessments remains limited by the small number of available studies.

Subgroup analyses based on geographical or cultural context were also not conducted due to the limited number of studies per region, which may restrict the ability to explore context-specific effects of interventions.

Assessment of publication bias was conducted using funnel plots and Egger’s test. However, due to the limited number of studies included in each analysis, the reliability of these methods may be reduced and results should be interpreted with caution.

Moreover, the exclusion of extreme effect sizes may have influenced the pooled estimates and contributed to the low levels of heterogeneity observed across analyses. This decision, while intended to improve stability, may introduce bias and should be considered when interpreting the results.

Furthermore, a formal GRADE assessment of the overall quality of evidence was not conducted due to the heterogeneity in study designs, interventions, and outcome measures, which limited the applicability of this framework.

Finally, a notable scarcity of nursing-led interventions was identified, particularly those adopting holistic approaches to mental health promotion. In addition, no studies specifically evaluated interventions focused on physical appearance care, highlighting an important gap in the current evidence base. Future research addressing this gap may contribute to a better understanding of the potential role of psychiatric nursing in stigma reduction and recovery-oriented care.

## 6. Conclusions

This systematic review and meta-analysis indicate that interventions targeting self-stigma in individuals with severe mental illness appear to be more effective when they integrate psychoeducation, cognitive restructuring strategies, and social support components. However, these findings should be interpreted with caution given the modest effect sizes and the methodological heterogeneity across the included studies, which limit the certainty and generalizability of the evidence.

The findings of this review reveal important gaps in the current evidence base. In particular, a notable scarcity of nursing-led interventions was identified, especially those adopting holistic approaches to mental health promotion. In addition, no studies specifically explored the potential role of physical appearance care in stigma reduction. Consequently, no direct evidence was identified regarding interventions targeting physical appearance, and any implications in this area should therefore be considered exploratory and subject to further empirical investigation. This absence of evidence highlights an important gap in the current literature and a potential area for future research.

Interventions led by psychiatric nursing professionals that incorporate holistic care perspectives and address identity-related aspects such as self-perception and body image may warrant further investigation. However, further empirical research is needed to determine their effectiveness and clinical applicability.

Future experimental studies are needed to evaluate the impact of such interventions across diverse clinical and cultural contexts, as well as longitudinal research to assess the sustainability of their effects over time. Future research should explore whether physical appearance-related components can contribute to recovery-oriented mental health care, as no direct empirical evidence was identified in the present review.

According to the Scottish Intercollegiate Guidelines Network (SIGN) grading framework, most included studies provide descriptive evidence at level 3 or clinical trial evidence ranging between levels 1− and 1+. Consequently, the available evidence supports a grade C recommendation for the implementation of anti-stigma intervention programs and the further development of nursing practices in this area. Overall, the available evidence suggests modest but potentially meaningful benefits, although conclusions should be interpreted cautiously due to limitations in the evidence base and the heterogeneity of included studies.

## Figures and Tables

**Table 1:** Table of scales used to assess each of the variables examined in the studies included in the meta-analysis.

Variables	Self-Stigma	Self-Esteem	Quality of Life	Self-Efficacy	Psychiatric Symptoms	Hope
Sudies	Scales					
Frączek-Cendrowska et al.	ISMI	RSES	SWLS	-	-	-
Tang et al.	PDD	-	-	-	BPRS	-
Drapalski et al.	ISMI	-	-	GSES	BSI	-
Yanos et al.	PDD	-	SQLS	-	BPRS	HHIS
Huang et al.	ISMI	RSES	-	-	BDI-II	HHIS
Li et al.	ISMI	SES	SQLS	-	BPRS	-
Modelli et al.	ISMI	RSES	SQLS	-	-	
Pijnenborg et al.	ISMI	RSES	MANSA	-	PANSS	-
Russinova et al.	ISMI	-	-	BOWC	-	WHS
Yanos et al.	ISMI	RSES	QLS	-	PANSS	BHS
Štrkalj Ivezić et al.	ISMI	-	-	BUES	-	-
Yılmaz and Kavak	ISMI	-	-	-	-	-

## Data Availability

No new data were created or analyzed in this study. Data sharing is not applicable to this article.

## Supplementary Materials

The following supporting information can be downloaded at: https://www.mdpi.com/article/10.3390/healthcare14131841/s1.

## References

[1] Haile Y.G., Habatmu K., Derese A., Gouse H., Lawrie S.M., Cella M., Alem A. (2022). Assessing cognition in people with severe mental disorders in low- and middle-income countries: A systematic review of assessment measures. Soc. Psychiatry Psychiatr. Epidemiol..

[2] World Health Organization (2003). Organization of Services for Mental Health.

[3] Kumari S., Joseph J., Singh B. (2023). Nurse-led brief psycho-education on self-stigma among clients with schizophrenia and affective disorders: -Solomon four-group design. Appl. Nurs. Res..

[4] American Psychiatric Association (2013). Diagnostic and Statistical Manual of Mental Disorders.

[5] World Health Organization (2018). International Classification of Diseases for Mortality and Morbidity Statistics.

[6] Dalky H.F. (2012). Mental Illness Stigma Reduction Interventions: Review of Intervention Trials. West J. Nurs. Res..

[7] Heijnders M., Van Der Meij S. (2006). The fight against stigma: An overview of stigma-reduction strategies and interventions. Psychol. Health Med..

[8] Link B.G., Phelan J.C. (2006). Stigma and its public health implications. Lancet.

[9] Madoz-Gúrpide A., Martín J.C.B., Sanmartín M.L., Yagüe E.G. (2017). Necesidad de un nuevo enfoque en la atención integral a los pacientes con trastorno mental grave treinta años después de la reforma psiquiátrica. Rev. Esp. Salud Publica.

[10] Richardson S., Lester L., Zhang G. (2012). Are Casual and Contract Terms of Employment Hazardous for Mental Health in Australia?. J. Ind. Relat..

[11] Liberman R.P., Kopelowicz A., Ventura J., Gutkind D. (2002). Operational criteria and factors related to recovery from schizophrenia. Int. Rev. Psychiatry.

[12] Goffman E. (1974). Stigma; Notes on the Management of Spoiled Identity.

[13] Corrigan P.W., Watson A.C., Ottati V. (2003). From whence comes mental illness stigma?. Int. J. Soc. Psychiatry.

[14] Corrigan P.W., Watson A.C., Barr L. (2006). The self-stigma of mental illness: Implications for self-esteem and self-efficacy. J. Soc. Clin. Psychol..

[15] Ebuenyi I.D., Syurina E.V., Bunders J.F.G., Regeer B.J. (2018). Barriers to and facilitators of employment for people with psychiatric disabilities in Africa: A scoping review. Glob. Health Action.

[16] Kaushik A., Kostaki E., Kyriakopoulos M. (2016). The stigma of mental illness in children and adolescents: A systematic review. Psychiatry Res..

[17] Michaels P.J., López M., Rüsch N., Corrigan P.W. (2017). Constructs and concepts comprising the stigma of mental illness. Psychol. Soc. Educ..

[18] Magliano L., Read J., Patalano M., Sagliocchi A., Oliviero N., D’Ambrosio A., Campitiello F., Zaccaro A., Guizzaro L., Cerrato F. (2017). Contrarrestar el estigma hacia las personas con esquizofrenia en el ámbito sanitario: Una experiencia piloto en una muestra de estudiantes italianos de medicina. Psychol. Soc. Educ..

[19] Bellack A.S. (2006). Scientific and consumer models of recovery in schizophrenia: Concordance, contrasts, and implications. Schizophr. Bull..

[20] Yanos P.T., DeLuca J.S., Roe D., Lysaker P.H. (2020). The impact of illness identity on recovery from severe mental illness: A review of the evidence. Psychiatry Res..

[21] Livingston J.D., Boyd J.E. (2010). Correlates and consequences of internalized stigma for people living with mental illness: A systematic review and meta-analysis. Soc. Sci. Med..

[22] Muñoz M., Sanz M., Pérez-Santos E., Quiroga M.d.l.Á (2011). Proposal of a socio-cognitive-behavioral structural equation model of internalized stigma in people with severe and persistent mental illness. Psychiatry Res..

[23] Díaz-Mandado O., Nieto-Moreno M., Montorio I., Periáñez J.A. (2015). Predictores de recuperación subjetiva en la esquizofrenia. Rev. Psicopatol. Psicol. Clin..

[24] Tsang H.W.H., Ching S.C., Tang K.H., Lam H.T., Law P.Y.Y., Wan C.N. (2016). Therapeutic intervention for internalized stigma of severe mental illness: A systematic review and meta-analysis. Schizophr. Res..

[25] Cobigo V., Stuart H. (2010). Social inclusion and mental health. Curr. Opin. Psychiatry.

[26] Corrigan P.W. (2016). Lessons learned from unintended consequences about erasing the stigma of mental illness. World Psychiatry.

[27] Baldwin M.L., Marcus S.C. (2011). Stigma, discrimination, and employment outcomes among persons with mental health disabilities. Work Accommodation and Retention in Mental Health.

[28] Wood L., Byrne R., Varese F., Morrison A.P. (2016). Psychosocial interventions for internalised stigma in people with a schizophrenia-spectrum diagnosis: A systematic narrative synthesis and meta-analysis. Schizophr. Res..

[29] Thorndike E.L. (1920). A constant error in psychological ratings. J. Appl. Psychol..

[30] Centro Cochrane Iberoamericano (2012). Manual Cochrane de Revisiones Sistemáticas de Intervenciones. Versión 5.1.

[31] Sterne J.A.C., Savović J., Page M.J., Elbers R.G., Blencowe N.S., Boutron I., Cates C.J., Cheng H.Y., Corbett M.S., Eldridge S.M. (2019). RoB 2: A revised tool for assessing risk of bias in randomised trials. BMJ.

[32] Page M.J., McKenzie J.E., Bossuyt P.M., Boutron I., Hoffmann T.C., Mulrow C.D., Shamseer L., Tetzlaff J.M., Akl E.A., Brennan S.E. (2021). Year: 2021. The PRISMA 2020 statement: An updated guideline for reporting systematic reviews. BMJ.

[33] Cabello J.B. (2015). Lectura Crítica de la Evidencia Clínica.

[34] Scottish Intercollegiate Guidelines Network (2019). SIGN 50: A Guideline Developer’s Handbook.

[35] Huang L.T., Liu C.Y., Yang C.Y. (2023). Narrative enhancement and cognitive therapy for perceived stigma of chronic schizophrenia: A multicenter randomized controlled trial study. Arch. Psychiatr. Nurs..

[36] Li J., Huang Y.G., Ran M.S., Fan Y., Chen W., Evans-Lacko S., Thornicroft G. (2018). Community-based comprehensive intervention for people with schizophrenia in Guangzhou, China: Effects on clinical symptoms, social functioning, internalized stigma and discrimination. Asian J. Psychiatr..

[37] Modelli A., Candal Setti V.P., van de Bilt M.T., Gattaz W.F., Loch A.A., Rössler W. (2021). Addressing Mood Disorder Diagnosis’ Stigma with an Honest, Open, Proud (HOP)-Based Intervention: A Randomized Controlled Trial. Front Psychiatry.

[38] Pijnenborg G.H.M., de Vos A.E., Timmerman M.E., Van der Gaag M., Sportel B.E., Arends J., Koopmans E., Van der Meer L., Aleman A. (2019). Social cognitive group treatment for impaired insight in psychosis: A multicenter randomized controlled trial. Schizophr. Res..

[39] Russinova Z., Mizock L., Bloch P. (2018). Photovoice as a tool to understand the experience of stigma among individuals with serious mental illnesses. Stigma Health.

[40] Tang M.W., Cheng Y., Zhang Y.H., Liu S.J. (2023). Effect of a Positive Psychology Expressive Writing on Stigma, Hope, Coping Style, and Quality of Life in Hospitalized Female Patients with Schizophrenia: A Randomized, Controlled Trial. Perspect. Psychiatr. Care.

[41] Tang Q., Yang S., Liu C., Li L., Chen X., Wu F., Huang X. (2021). Effects of mindfulness-based cognitive therapy on stigma in female patients with schizophrenia. Front. Psychiatry.

[42] Yanos P.T., Lysaker P.H., Silverstein S.M., Vayshenker B., Gonzales L., West M.L., Roe D. (2019). A randomized-controlled trial of treatment for self-stigma among persons diagnosed with schizophrenia-spectrum disorders. Soc. Psychiatry Psychiatr. Epidemiol..

[43] Frączek-Cendrowska K., Świtaj P., Stefaniak I. (2024). Evaluation of the Effectiveness of a Group CBT-Based Intervention Aiming to Reduce Self-Stigma and Improve Recovery-Related Outcomes in People with Severe Mental Disorders: Randomised Controlled Trial. Psychiatr. Q..

[44] Drapalski A.L., Lucksted A., Brown C.H., Fang L.J. (2021). Outcomes of Ending Self-Stigma, a Group Intervention to Reduce Internalized Stigma, Among Individuals with Serious Mental Illness. Psychiatr. Serv..

[45] Çapar M., Kavak F. (2019). Effect of internalized stigma on functional recovery in patients with schizophrenia. Perspect. Psychiatr. Care.

[46] Grover S., Hazari N., Aneja J., Chakrabarti S., Avasthi A. (2016). Stigma and its correlates among patients with bipolar disorder: A study from a tertiary care hospital of North India. Psychiatry Res..

[47] Kim W.J., Song Y.J., Ryu H.S., Ryu V., Kim J.M., Ha R.Y., Lee S.J., Namkoong K., Ha K., Cho H.-S. (2015). Internalized stigma and its psychosocial correlates in Korean patients with serious mental illness. Psychiatry Res..

[48] Li X.H., Zhang T.M., Yau Y.Y., Wang Y.Z., Wong Y.L.I., Yang L., Tian X.-L., Chan C.L.-W., Ran M.-S. (2021). Peer-to-peer contact, social support and self-stigma among people with severe mental illness in Hong Kong. Int. J. Soc. Psychiatry.

[49] Morgades-Bamba C.I., Fuster-Ruizdeapodaca M.J., Molero F. (2019). The impact of internalized stigma on the well-being of people with Schizophrenia. Psychiatry Res..

[50] Ran M.S., Zhang T.M., Wong I.Y.L., Yang X., Liu C.C., Liu B., Luo W., Kuang W.-H., Thornicroft G., Chan C.L.-W. (2018). Internalized stigma in people with severe mental illness in rural China. Int. J. Soc. Psychiatry.

[51] Violeau L., Dudilot A., Roux S., Prouteau A. (2020). How internalised stigma reduces self-esteem in schizophrenia: The crucial role of off-line metacognition. Cogn. Neuropsychiatry.

[52] Cullen B.A.M., Mojtabai R., Bordbar E., Everett A., Nugent K.L., Eaton W.W. (2017). Social network, recovery attitudes and internal stigma among those with serious mental illness. Int. J. Soc. Psychiatry.

[53] Cunningham K.C., Lucksted A. (2017). Social Cognition, Internalized Stigma, and Recovery Orientation among Adults with Serious Mental Illness. Psychiatr. Rehabil. J..

[54] Grover S., Avasthi A., Singh A., Dan A., Neogi R., Kaur D., Lakdawala B., Rozatkar A.R., Nebhinani N., Patra S. (2017). Stigma experienced by patients with severe mental disorders: A nationwide multicentric study from India. Psychiatry Res..

[55] Hack S.M., Muralidharan A., Brown C.H., Drapalski A.L., Lucksted A.A. (2020). Stigma and discrimination as correlates of mental health treatment engagement among adults with serious mental illness. Psychiatr. Rehabil. J..

[56] Liu X., Yin M., Li Z., Wang D. (2024). Psychosocial Correlates of Internalized Stigma Among Chinese Individuals with Severe Mental Illness. J. Psychosoc. Nurs. Ment. Health Serv..

[57] Ouali U., Jouini L., Ouertani H., Jomli R., Nacef F. (2020). The stigma of severe mental illness: Beliefs and experiences of tunisian patients. Tunis. Medicale.

[58] Villotti P., Zaniboni S., Corbière M., Guay S., Fraccaroli F. (2018). Reducing perceived stigma: Work integration of people with severe mental disorders in Italian social enterprise. Psychiatr. Rehabil. J..

[59] Ma N., Chen R., Bai Y., Zhang W., Chen Z., Zhou J., Cao Y., Wen L., Chen X., Zhan X. (2024). A longitudinal study on the effects of social support on self-stigma, psychiatric symptoms, and personal and social functioning in community patients with severe mental illnesses in China. Int. J. Soc. Psychiatry.

[60] Yılmaz E., Kavak F. (2020). Effects of Mindfulness-Based Psychoeducation on the Internalized Stigmatization Level of Patients with Schizophrenia. Clin. Nurs. Res..

[61] Strkalj Ivezic S., Sesar M.A., Muzinic L. (2017). Effects of a group psychoeducation program on self-stigma, empowerment and perceived discrimination of persons with schizophrenia. Psychiatr. Danub..

[62] Alonso M., Guillén A.I., Muñoz M. (2019). Interventions to Reduce Internalized Stigma in individuals with Mental Illness: A Systematic Review. Span. J. Psychol..

[63] Russinova Z., Gidugu V., Bloch P., Restrepo-Toro M., Sally Rogers E. (2018). Empowering individuals with psychiatric disabilities to work: Results of a randomized trial. Psychiatr. Rehabil. J..

[64] Villotti P., Corbière M., Dewa C.S., Fraccaroli F., Sultan-Taïeb H., Zaniboni S., Lecomte T. (2018). A serial mediation model of workplace social support on work productivity: The role of self-stigma and job tenure self-efficacy in people with severe mental disorders. Disabil. Rehabil..

