# Supplementary material for: Effectiveness of Non-Pharmacological Interventions for Reducing Self-Stigma in Adults with Severe Mental Illness: A Systematic Review and Meta-Analysis
Source: Healthcare (Basel). 2026 Jun 24;14(13):1841. doi: 10.3390/healthcare14131841 (PMC13361077; doi:10.3390/healthcare14131841)
Supplement: Supplementary file 1 [file healthcare-14-01841-s001.zip › healthcare-4310454-supplementary.pdf]

Supplementary File S1. Detailed Search Strategies

This supplementary file presents the electronic search strategies used across all databases included in this systematic review and meta-analysis. The strategies were reconstructed based on the original search approach and adapted to the syntax and indexing of each database to ensure transparency and facilitate reproducibility.

PubMed (via NCBI)

Search date: May 2025

Search strategy:

("Mental Disorders"[MeSH] OR "severe mental disorder")

AND

("Social Stigma"[MeSH] OR stigma OR "stigma reduction" OR hygiene OR cosmetics OR clothing)

Filters applied:

• Language: English, Spanish

• Publication date: Last 10 years

• Population: Adults (≥18 years)

Web of Science

Search date: May 2025

Search strategy:

TS= ("Mental Disorders" OR "severe mental disorder")

AND

TS= ("Social Stigma" OR stigma OR "stigma reduction" OR hygiene OR cosmetics OR clothing)

Filters applied:

• Language: English, Spanish

• Document type: Articles

• Timespan: Last 10 years

Cochrane Library

Search date: May 2025

Search strategy:

("Mental Disorders" OR "severe mental disorder")

AND

("Social Stigma" OR stigma OR hygiene OR cosmetics OR clothing)

Filters applied:

• Study type: Trials and reviews

• Language: English, Spanish

• Publication date: Last 10 years

EBSCOhost databases (APA PsycInfo, CINAHL, SocINDEX, etc.)

Search date: May 2025

Search strategy:

("Mental Disorders" OR "severe mental disorder")

AND

("Social Stigma" OR stigma OR "stigma reduction" OR hygiene OR cosmetics OR clothing)

Filters applied:

• Peer-reviewed articles

• Language: English, Spanish

• Publication date: Last 10 years

Virtual Health Library (BVS)

Search date: May 2025

Search strategy:

("Trastornos Mentales" OR "Enfermedad Mental Grave")

AND

("Estigma Social" OR higiene OR cosméticos OR vestimenta)

Filters applied:

• Language: Spanish, English

• Publication date: Last 10 years

Supplementary File S2 – Publication Bias Assessment

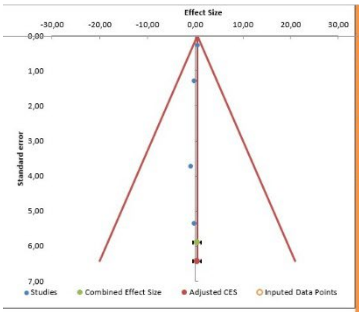

Funnel Plot (with trim-and-fill)

Combined effect size	Observed
Effect Size	0,44
SE	0,26
CI Lower limit	-0,40
CI Upper limit	1,27
PI Lower limit	-0,40
PI Upper limit	1,27

Heterogeneity	Adjusted
Q	0,42
p_Q	0,936
I^2	0,00%
T^2	0,00
T	0,00

Combined effect size	Adjusted
Effect Size	0,44
SE	0,26
CI Lower limit	-0,40
CI Upper limit	1,27
PI Lower limit	-0,40
PI Upper limit	1,27

Trim and Fill	On
Estimator for missing studies	Leftmost Run/Rightmost Run
Search from mean	Left
Number of imputed studies	0

Egger Regression and Begg & Mazumdar Rank Correlation

Egger Regression	Estimate	SE	CI LL	CI UL
Intercept	-0,37	0,15	-0,85	0,11
Slope	0,56	0,08	0,31	0,82

t test	-2,45
p-value	0,134

Begg & Mazumdar's rank correlation test	
Δx-y	0
Kendall's Tau a	0,00
z-value	0,00
p-value	0,500

Study name	Standardized residual
Yanos et al.	-0,38
Huang et al	-0,12
Russinova e	-0,51
Yanos et al.	0,62

Standardized Residual Histogram

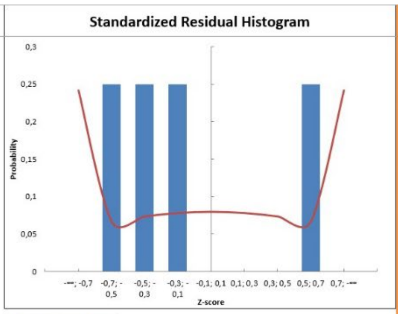

Bin	Proportion	Probability
-∞; -0,7	0,00	0,24
-0,7; -0,5	0,25	0,07
-0,5; -0,3	0,25	0,07
-0,3; -0,1	0,25	0,08
-0,1; 0,1	0,00	0,08
0,1; 0,3	0,00	0,08
0,3; 0,5	0,00	0,07
0,5; 0,7	0,25	0,07
0,7; -∞	0,00	0,24

* upper bound of bin is inclusive

Publication bias analysis for the quality-of-life

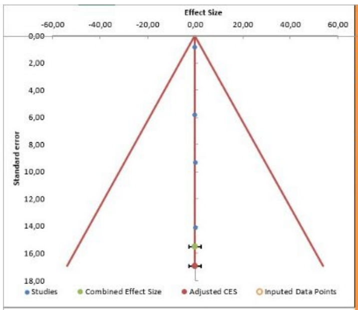

Funnel Plot (with trim-and-fill)

Combined effect size	Observed
Effect Size	-0,18
SE	0,81
CI Lower limit	-2,75
CI Upper limit	2,39
PI Lower limit	-2,75
PI Upper limit	2,39

Heterogeneity	Adjusted
Q	0,00
p_Q	1,000
I^2	0,00%
T^2	0,00
T	0,00

Combined effect size	Adjusted
Effect Size	-0,18
SE	0,81
CI Lower limit	-2,75
CI Upper limit	2,39
PI Lower limit	-2,75
PI Upper limit	2,39

Trim and Fill	On
Estimator for missing studies	Leftmost Run/Rightmost Run
Search from mean	Right
Number of imputed studies	0

Egger Regression and Begg & Mazumdar Rank Correlation

Egger Regression	Estimate	SE	CI LL	CI UL
Intercept	0,00	0,02	-0,04	0,05
Slope	-0,19	0,02	-0,27	-0,11

t test	0,22
p-value	0,848

Begg & Mazumdar's rank correlation test	
Δx-y	4
Kendall's Tau a	0,67
z-value	1,36
p-value	0,087

Study name	Standardized residual
Frączek-Cen	-0,03
Li et al. (201	0,02
Pijnenborg	0,01
Yanos et al.	0,01

Standardized Residual Histogram

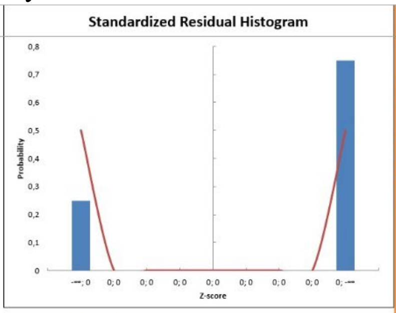

Bin	Proportion	Probability
-∞; 0	0,25	0,50
0; 0	0,00	0,00
0; 0	0,00	0,00
0; 0	0,00	0,00
0; 0	0,00	0,00
0; 0	0,00	0,00
0; 0	0,00	0,00
0; 0	0,00	0,00
0; -∞	0,75	0,50

* upper bound of bin is inclusive

Publication bias analysis for the self-efficacy

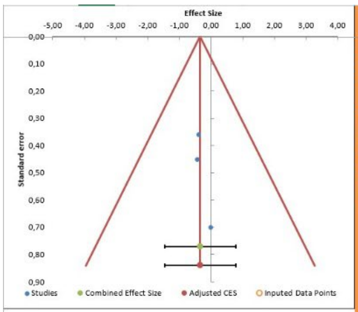

Funnel Plot (with trim-and-fill)

Combined effect size	Observed
Effect Size	-0,35
SE	0,26
CI Lower limit	-1,47
CI Upper limit	0,78
PI Lower limit	-1,47
PI Upper limit	0,78

Heterogeneity	Adjusted
Q	0,29
p_Q	0,866
I^2	0,00%
T^2	0,00
T	0,00

Combined effect size	Adjusted
Effect Size	-0,35
SE	0,26
CI Lower limit	-1,47
CI Upper limit	0,78
PI Lower limit	-1,47
PI Upper limit	0,78

Trim and Fill	On
Estimator for missing studies	Leftmost Run/Rightmost Run
Search from mean	Right
Number of imputed studies	0

Egger Regression and Begg & Mazumdar Rank Correlation

Egger Regression	Estimate	SE	CI LL	CI UL
Intercept	1,10	0,57	-1,34	3,54
Slope	-0,83	0,26	-1,93	0,28

t test	1,93
p-value	0,304

Begg & Mazumdar's rank correlation test	
Δx-y	1
Kendall's Tau a	0,33
z-value	0,52
p-value	0,301

Study name	Standardized residual
Drapalski e	0,53
Russinova e	-0,21
Štrkalj Ivezi	-0,17

Standardized Residual Histogram

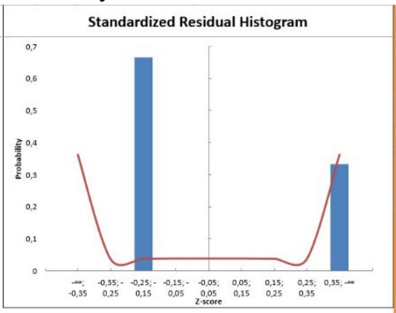

Bin	Proportion	Probability
-∞; -0,35	0,00	0,36
-0,35; -0,2	0,00	0,04
-0,25; -0,1	0,67	0,04
-0,15; -0,0	0,00	0,04
-0,05; 0,05	0,00	0,04
0,05; 0,15	0,00	0,04
0,15; 0,25	0,00	0,04
0,25; 0,35	0,00	0,04
0,35; -∞	0,33	0,36

* upper bound of bin is inclusive

Publication bias analysis for the self-esteem

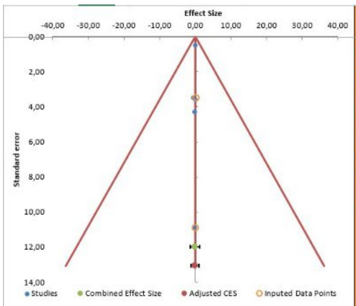

Funnel Plot (with trim-and-fill)

Combined effect size	Observed
Effect Size	-0,05
SE	0,48
CI Lower limit	-1,38
CI Upper limit	1,28
PI Lower limit	-1,38
PI Upper limit	1,28

Heterogeneity	Adjusted
Q	0,03
p_Q	1,000
I^2	0,00%
T^2	0,00
T	0,00

Combined effect size	Adjusted
Effect Size	-0,04
SE	0,47
CI Lower limit	-1,20
CI Upper limit	1,12
PI Lower limit	-1,20
PI Upper limit	1,12

Trim and Fill	On
Estimator for missing studies	Leftmost Run/Rightmost Run
Search from mean	Right
Number of imputed studies	2

Egger Regression	Estimate	SE	CI LL	CI UL
Intercept	-0,04	0,04	-0,14	0,06
Slope	-0,02	0,04	-0,13	0,08

t test	-1,10
p-value	0,350

Begg & Mazumdar's rank correlation test	
Δx-y	-2
Kendall's Tau a	-0,20
z-value	-0,49
p-value	0,312

Egger Regression and Begg & Mazumdar Rank Correlation

Study name	Standardized residual
Frączek-Cen	-0,13
Pijnenborg	-0,01
Huang et al	-0,02
Li et al. (201	0,02
Yanos et al.	0,08

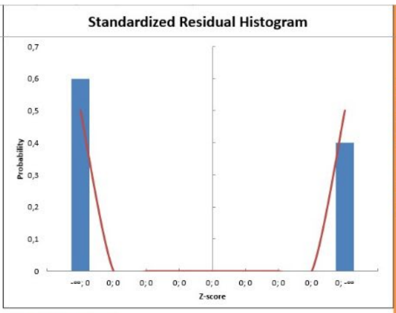

Bin	Proportion	Probability
-∞; 0	0,60	0,50
0; 0	0,00	0,00
0; 0	0,00	0,00
0; 0	0,00	0,00
0; 0	0,00	0,00
0; 0	0,00	0,00
0; 0	0,00	0,00
0; 0	0,00	0,00
0; -∞	0,40	0,50

* upper bound of bin is inclusive

Standardized Residual Histogram

Publication bias analysis for the self-stigma

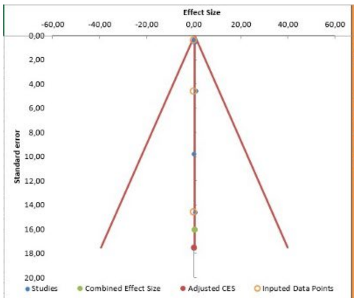

Funnel Plot (with trim-and-fill)

Combined effect size	Observed
Effect Size	0,34
SE	0,15
CI Lower limit	0,00
CI Upper limit	0,69
PI Lower limit	0,00
PI Upper limit	0,69

Heterogeneity	Adjusted
Q	1,85
p_Q	1,000
I^2	0,00%
T^2	0,00
T	0,00

Combined effect size	Adjusted
Effect Size	0,30
SE	0,14
CI Lower limit	-0,01
CI Upper limit	0,61
PI Lower limit	-0,01
PI Upper limit	0,61

Trim and Fill	On
Estimator for missing studies	Leftmost Run/Rightmost Run
Search from mean	Left
Number of imputed studies	3

Egger Regression	Estimate	SE	CI LL	CI UL
Intercept	0,06	0,22	-0,45	0,56
Slope	0,32	0,11	0,08	0,56

t test	0,26
p-value	0,799

Begg & Mazumdar's rank correlation test	
Δx-y	5
Kendall's Tau a	0,11
z-value	0,45
p-value	0,327

Egger Regression and Begg & Mazumdar Rank Correlation

Study name	Standardized residual
Frączek-Cen	-0,04
Drapalski e	-0,25
Huang et al	-0,46
Li et al. (201	-0,54
Pijnenborg	-0,02
Russinova e	0,54
Yanos et al.	0,11
Štrkalj Ivezi	0,70
Yılmaz y Kav	0,02
Tang et al. (	0,10

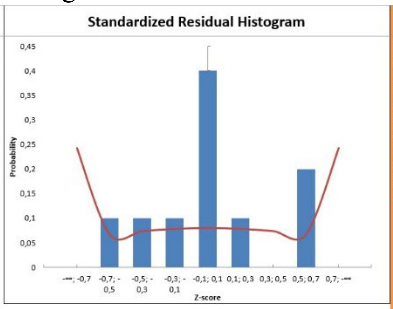

Bin	Proportion	Probability
-∞; -0,7	0,00	0,24
-0,7; -0,5	0,10	0,07
-0,5; -0,3	0,10	0,07
-0,3; -0,1	0,10	0,08
-0,1; 0,1	0,40	0,08
0,1; 0,3	0,10	0,08
0,3; 0,5	0,00	0,07
0,5; 0,7	0,20	0,07
0,7; -∞	0,00	0,24

* upper bound of bin is inclusive

Standardized Residual Histogram

Publication bias analysisfor the clinical symptomatology

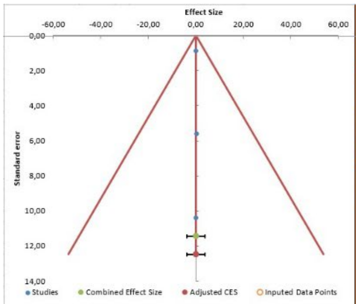

Funnel Plot (with trim-and-fill)

Combined effect size	Observed
Effect Size	0,05
SE	0,87
CI Lower limit	-3,67
CI Upper limit	3,78
PI Lower limit	-3,67
PI Upper limit	3,78

Heterogeneity	Adjusted
Q	0,01
p_Q	0,997
I^2	0,00%
T^2	0,00
T	0,00

Combined effect size	Adjusted
Effect Size	0,05
SE	0,87
CI Lower limit	-3,67
CI Upper limit	3,78
PI Lower limit	-3,67
PI Upper limit	3,78

Trim and Fill	On
Estimator for missing studies	Leftmost Run/Rightmost Run
Search from mean	Left
Number of imputed studies	0

Egger Regression	Estimate	SE	CI LL	CI UL
Intercept	0,03	0,05	-0,18	0,24
Slope	0,02	0,07	-0,30	0,34

t test	0,64
p-value	0,639

Begg & Mazumdar's rank correlation test	
Δx-y	1
Kendall's Tau a	0,33
z-value	0,52
p-value	0,301

Egger Regression and Begg & Mazumdar Rank Correlation

Study name	Standardized residual
Huang et al	-0,01
Pijnenborg	0,07
Yanos et al.	-0,06

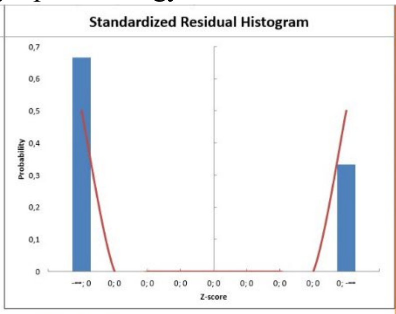

Bin	Proportion	Probability
-∞; 0	0,67	0,50
0; 0	0,00	0,00
0; 0	0,00	0,00
0; 0	0,00	0,00
0; 0	0,00	0,00
0; 0	0,00	0,00
0; 0	0,00	0,00
0; 0	0,00	0,00
0; -∞	0,33	0,50

* upper bound of bin is inclusive

Standardized Residual Histogram

Supplementary File S3 Leave-One-Out Sensitivity Analysis for Self-Stigma Meta-Analysis

Input Frączek-Cendrowska et al. (2024)

#	Study name	Include study	Effect size	Standard error	Number of observations (for CIs)	Sufficient data	Subgroup	Moderator
	Frączek-Cen	No	0,33	0,30	77	Yes	AA	15
1	Drapalski e	Yes	0,25	0,40	216	Yes	AA	16
2	Huang et al	Yes	0,14	0,47	86	Yes	AA	13
3	Li et al. (201	Yes	0,16	0,37	327	Yes	AA	18
4	Pijnenborg (	Yes	0,18	9,80	121	Yes	BB	20
5	Russinova e	Yes	0,57	0,44	46	Yes	BB	14
6	Yanos et al.	Yes	0,40	0,57	117	Yes	AA	19
7	Štrkalj Ivezi	Yes	0,61	0,41	80	Yes	AA	13
8	Yılmaz y Kav	Yes	0,66	14,63	69	Yes	BB	19
9	Tang et al. (	Yes	0,78	4,60	59	Yes	AA	22

Forest plot Frączek-Cendrowska et al. (2024)

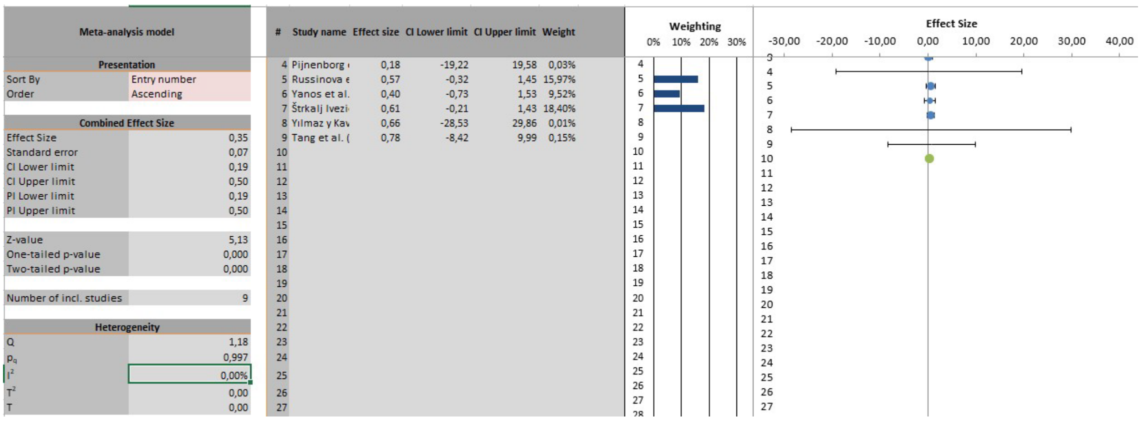

Input Huang et al. (2023)

#	Study name	Include study	Effect size	Standard error	Number of observations (for CIs)	Sufficient data	Subgroup	Moderator
1	Frączek-Cen	Yes	0,33	0,30	77	Yes	AA	15
2	Drapalski e	Yes	0,25	0,40	216	Yes	AA	16
	Huang et al	No	0,14	0,47	86	Yes	AA	13
3	Li et al. (201	Yes	0,16	0,37	327	Yes	AA	18
4	Pijnenborg (	Yes	0,18	9,80	121	Yes	BB	20
5	Russinova e	Yes	0,57	0,44	46	Yes	BB	14
6	Yanos et al.	Yes	0,40	0,57	117	Yes	AA	19
7	Štrkalj Ivezi	Yes	0,61	0,41	80	Yes	AA	13
8	Yılmaz y Kav	Yes	0,66	14,63	69	Yes	BB	19
9	Tang et al. (	Yes	0,78	4,60	59	Yes	AA	22

Forest plot Huang et al. (2023)

Meta-analysis model	
**Presentation**	
Sort By	Entry number
Order	Ascending
**Combined Effect Size**	
Effect Size	0,37
Standard error	0,06
CI Lower limit	0,24
CI Upper limit	0,50
PI Lower limit	0,24
PI Upper limit	0,50
Z-value	6,56
One-tailed p-value	0,000
Two-tailed p-value	0,000
Number of incl. studies	9
**Heterogeneity**	
Q	0,98
p_q	0,998
I^2	0,00%
T^2	0,00
T	0,00

#	Study name	Effect size	CI Lower limit	CI Upper limit	Weight
4	Pijnenborg	0,18	-19,22	19,58	0,03%
5	Russinova	0,57	-0,32	1,45	13,27%
6	Yanos et al.	0,40	-0,73	1,53	7,91%
7	Štrkalj Ivezi	0,61	-0,21	1,43	15,28%
8	Yılmaz y Kav	0,66	-28,53	29,86	0,01%
9	Tang et al. (	0,78	-8,42	9,99	0,12%

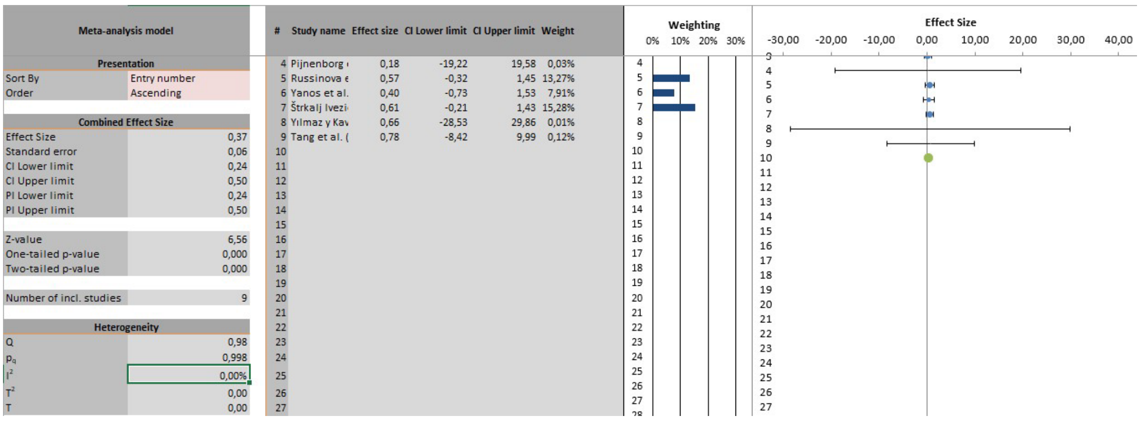

Input Drapalski et al. (2021)

#	Study name	Include study	Effect size	Standard error	Number of observations (for CIs)	Sufficient data	Subgroup	Moderator
1	Frączek-Cen	Yes	0,33	0,30	77	Yes	AA	15
	Drapalski e	No	0,25	0,40	216	Yes	AA	16
2	Huang et al	Yes	0,14	0,47	86	Yes	AA	13
3	Li et al. (201	Yes	0,16	0,37	327	Yes	AA	18
4	Pijnenborg	Yes	0,18	9,80	121	Yes	BB	20
5	Russinova	Yes	0,57	0,44	46	Yes	BB	14
6	Yanos et al.	Yes	0,40	0,57	117	Yes	AA	19
7	Štrkalj Ivezi	Yes	0,61	0,41	80	Yes	AA	13
8	Yılmaz y Kav	Yes	0,66	14,63	69	Yes	BB	19
9	Tang et al. (	Yes	0,78	4,60	59	Yes	AA	22

Forest plot Drapalski et al. (2021)

Meta-analysis model	
**Presentation**	
Sort By	Entry number
Order	Ascending
**Combined Effect Size**	
Effect Size	0,36
Standard error	0,06
CI Lower limit	0,22
CI Upper limit	0,50
PI Lower limit	0,22
PI Upper limit	0,50
Z-value	5,85
One-tailed p-value	0,000
Two-tailed p-value	0,000
Number of incl. studies	9
**Heterogeneity**	
Q	1,12
p_q	0,997
I^2	0,00%
T^2	0,00
T	0,00

#	Study name	Effect size	CI Lower limit	CI Upper limit	Weight
4	Pijnenborg	0,18	-19,22	19,58	0,03%
5	Russinova	0,57	-0,32	1,45	13,89%
6	Yanos et al.	0,40	-0,73	1,53	8,27%
7	Štrkalj Ivezi	0,61	-0,21	1,43	15,99%
8	Yılmaz y Kav	0,66	-28,53	29,86	0,01%
9	Tang et al. (	0,78	-8,42	9,99	0,13%

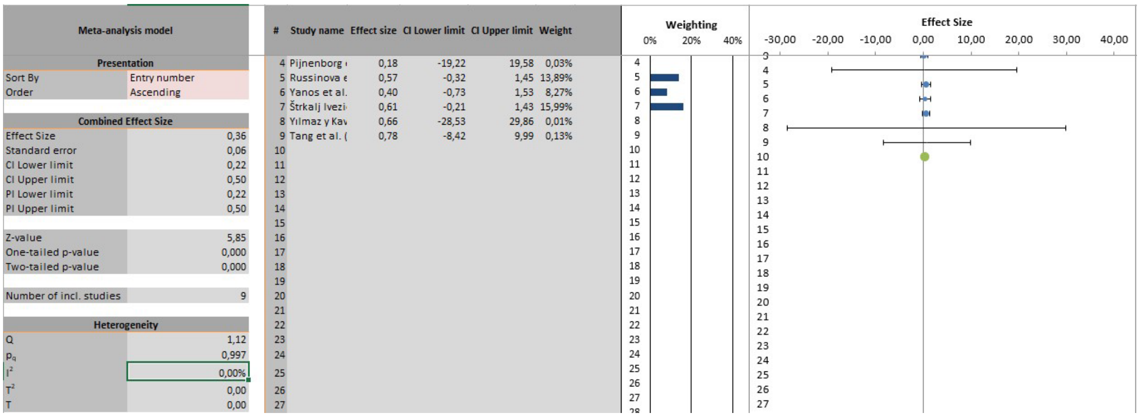

Input Li et al. (2018)

#	Study name	Include study	Effect size	Standard error	Number of observations (for CIs)	Sufficient data	Subgroup	Moderator
1	Frączek-Cen	Yes	0,33	0,30	77	Yes	AA	15
2	Drapalski e	Yes	0,25	0,40	216	Yes	AA	16
3	Huang et al	Yes	0,14	0,47	86	Yes	AA	13
	Li et al. (20	No	0,16	0,37	327	Yes	AA	18
4	Pijnenborg	Yes	0,18	9,80	121	Yes	BB	20
5	Russinova	Yes	0,57	0,44	46	Yes	BB	14
6	Yanos et al.	Yes	0,40	0,57	117	Yes	AA	19
7	Štrkalj Ivezi	Yes	0,61	0,41	80	Yes	AA	13
8	Yılmaz y Kav	Yes	0,66	14,63	69	Yes	BB	19
9	Tang et al. (	Yes	0,78	4,60	59	Yes	AA	22

Forest plot Li et al. (2018)

Meta-analysis model	
**Presentation**	
Sort By	Entry number
Order	Ascending
**Combined Effect Size**	
Effect Size	0,38
Standard error	0,06
CI Lower limit	0,25
CI Upper limit	0,51
PI Lower limit	0,25
PI Upper limit	0,51
Z-value	6,83
One-tailed p-value	0,000
Two-tailed p-value	0,000
Number of incl. studies	9
**Heterogeneity**	
Q	0,90
p_q	0,999
I^2	0,00%
T^2	0,00
T	0,00

#	Study name	Effect size	CI Lower limit	CI Upper limit	Weight
4	Pijnenborg (	0,18	-19,22	19,58	0,03%
5	Russinova e	0,57	-0,32	1,45	14,29%
6	Yanos et al.	0,40	-0,73	1,53	8,52%
7	Štrkalj Ivezi	0,61	-0,21	1,43	16,46%
8	Yılmaz y Kav	0,66	-28,53	29,86	0,01%
9	Tang et al. (	0,78	-8,42	9,99	0,13%

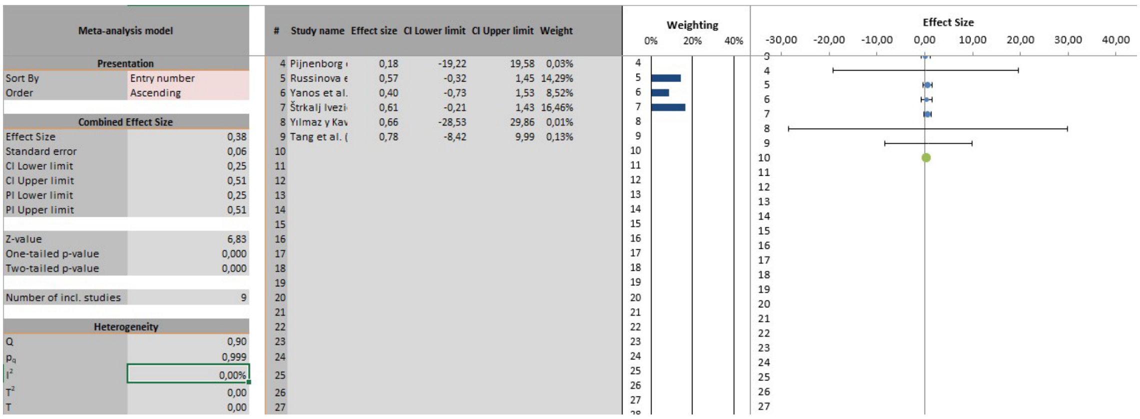

Input Pijnenborg et al. (2019)

#	Study name	Include study	Effect size	Standard error	Number of observations (for CIs)	Sufficient data	Subgroup	Moderator
1	Frączek-Cen	Yes	0,33	0,30	77	Yes	AA	15
2	Drapalski e	Yes	0,25	0,40	216	Yes	AA	16
3	Huang et al	Yes	0,14	0,47	86	Yes	AA	13
4	Li et al. (201	Yes	0,16	0,37	327	Yes	AA	18
	Pijnenborg	No	0,18	9,80	121	Yes	BB	20
5	Russinova e	Yes	0,57	0,44	46	Yes	BB	14
6	Yanos et al.	Yes	0,40	0,57	117	Yes	AA	19
7	Štrkalj Ivezi	Yes	0,61	0,41	80	Yes	AA	13
8	Yılmaz y Kav	Yes	0,66	14,63	69	Yes	BB	19
9	Tang et al. (	Yes	0,78	4,60	59	Yes	AA	22

Forest plot Pijnenborg et al. (2019)

Meta-analysis model	
**Presentation**	
Sort By	Entry number
Order	Ascending
**Combined Effect Size**	
Effect Size	0,34
Standard error	0,06
CI Lower limit	0,21
CI Upper limit	0,48
PI Lower limit	0,21
PI Upper limit	0,48
Z-value	5,88
One-tailed p-value	0,000
Two-tailed p-value	0,000
Number of incl. studies	9
**Heterogeneity**	
Q	1,19
p_q	0,997
I^2	0,00%
T^2	0,00
T	0,00

#	Study name	Effect size	CI Lower limit	CI Upper limit	Weight
4	Li et al. (201	0,16	-0,57	0,89	16,82%
5	Russinova e	0,57	-0,32	1,45	11,89%
6	Yanos et al.	0,40	-0,73	1,53	7,09%
7	Štrkalj Ivezi	0,61	-0,21	1,43	13,70%
8	Yılmaz y Kav	0,66	-28,53	29,86	0,01%
9	Tang et al. (	0,78	-8,42	9,99	0,11%

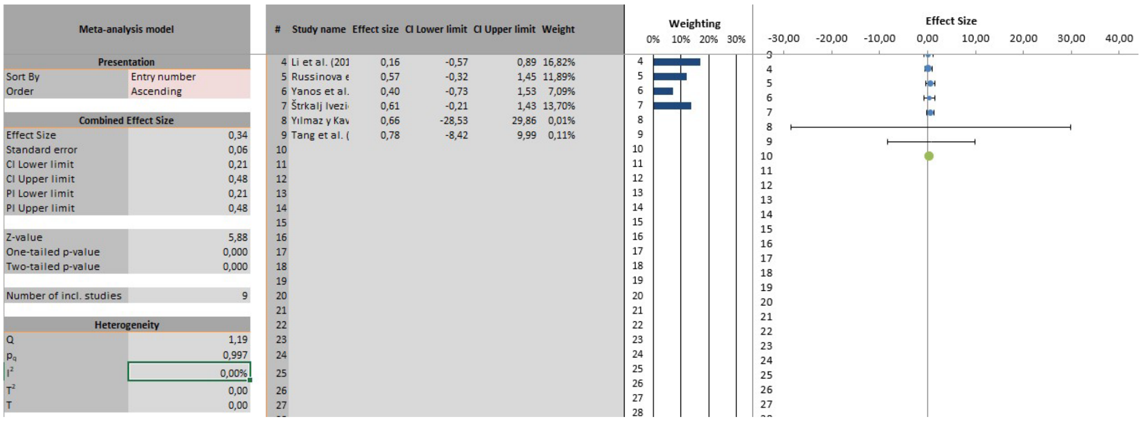

Input Russinova et al. (2018)

#	Study name	Include study	Effect size	Standard error	Number of observations (for CIs)	Sufficient data	Subgroup	Moderator
1	Frączek-Cen	Yes	0,33	0,30	77	Yes	AA	15
2	Drapalski e	Yes	0,25	0,40	216	Yes	AA	16
3	Huang et al	Yes	0,14	0,47	86	Yes	AA	13
4	Li et al. (201	Yes	0,16	0,37	327	Yes	AA	18
5	Pijnenborg (	Yes	0,18	9,80	121	Yes	BB	20
	Russinova e	No	0,57	0,44	46	Yes	BB	14
6	Yanos et al.	Yes	0,40	0,57	117	Yes	AA	19
7	Štrkalj Ivezi	Yes	0,61	0,41	80	Yes	AA	13
8	Yılmaz y Kav	Yes	0,66	14,63	69	Yes	BB	19
9	Tang et al. (	Yes	0,78	4,60	59	Yes	AA	22

Forest plot Russinova et al. (2018)

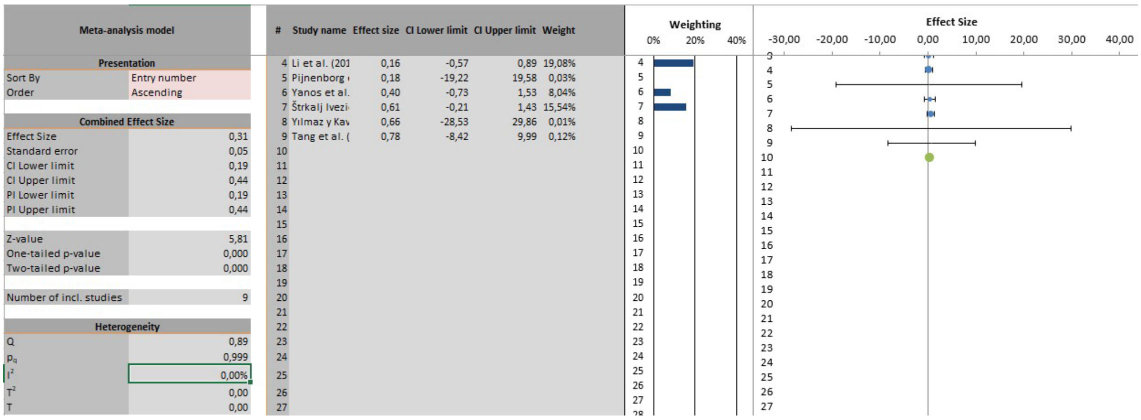

Input Štrkalj Ivezić et al. (2017)

#	Study name	Include study	Effect size	Standard error	Number of observations (for CIs)	Sufficient data	Subgroup	Moderator
1	Frączek-Cen	Yes	0,33	0,30	77	Yes	AA	15
2	Drapalski e	Yes	0,25	0,40	216	Yes	AA	16
3	Huang et al	Yes	0,14	0,47	86	Yes	AA	13
4	Li et al. (201	Yes	0,16	0,37	327	Yes	AA	18
5	Pijnenborg	Yes	0,18	9,80	121	Yes	BB	20
6	Russinova e	Yes	0,57	0,44	46	Yes	BB	14
7	Yanos et al.	Yes	0,40	0,57	117	Yes	AA	19
	Štrkalj Ivezi	No	0,61	0,41	80	Yes	AA	13
8	Yılmaz y Kav	Yes	0,66	14,63	69	Yes	BB	19
9	Tang et al. (	Yes	0,78	4,60	59	Yes	AA	22

Forest plot Štrkalj Ivezić et al. (2017)

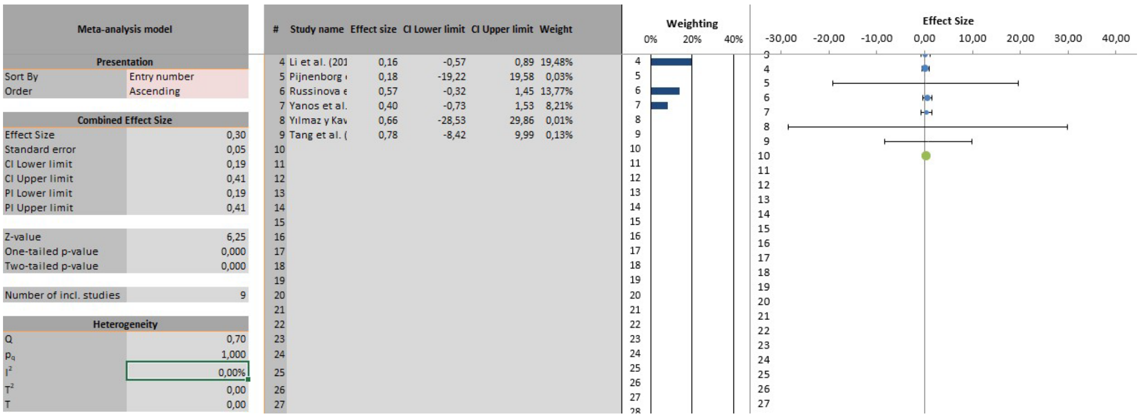

Input Tang et al. (2021)

#	Study name	Include study	Effect size	Standard error	Number of observations (for CIs)	Sufficient data	Subgroup	Moderator
1	Frączek-Cen	Yes	0,33	0,30	77	Yes	AA	15
2	Drapalski e	Yes	0,25	0,40	216	Yes	AA	16
3	Huang et al	Yes	0,14	0,47	86	Yes	AA	13
4	Li et al. (201	Yes	0,16	0,37	327	Yes	AA	18
5	Pijnenborg	Yes	0,18	9,80	121	Yes	BB	20
6	Russinova e	Yes	0,57	0,44	46	Yes	BB	14
7	Yanos et al.	Yes	0,40	0,57	117	Yes	AA	19
8	Štrkalj Ivezi	Yes	0,61	0,41	80	Yes	AA	13
9	Yılmaz y Kav	Yes	0,66	14,63	69	Yes	BB	19
	Tang et al. (	No	0,78	4,60	59	Yes	AA	22

Forest plot Tang et al. (2021)

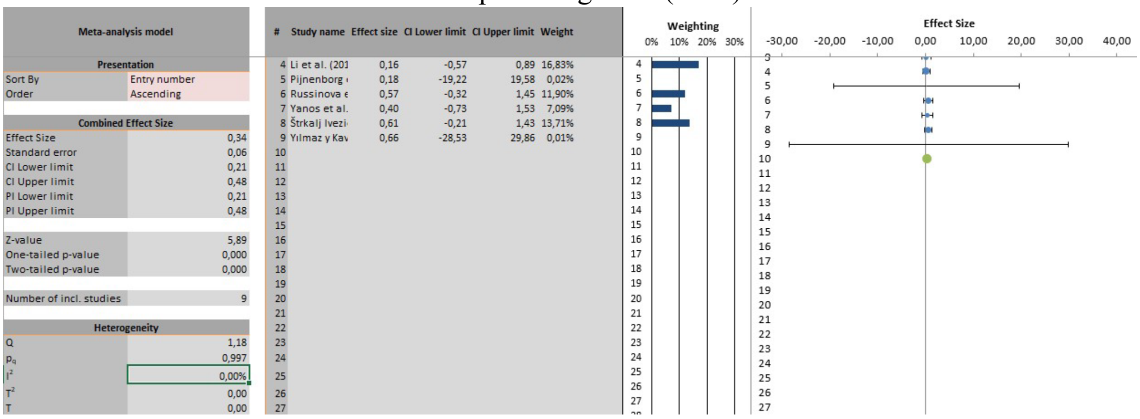

Input Yanos et al. (2019)

#	Study name	Include study	Effect size	Standard error	Number of observations (for CIs)	Sufficient data	Subgroup	Moderator
1	Frączek-Cen	Yes	0,33	0,30	77	Yes	AA	15
2	Drapalski e	Yes	0,25	0,40	216	Yes	AA	16
3	Huang et al	Yes	0,14	0,47	86	Yes	AA	13
4	Li et al. (201	Yes	0,16	0,37	327	Yes	AA	18
5	Pijnenborg	Yes	0,18	9,80	121	Yes	BB	20
6	Russinova e	Yes	0,57	0,44	46	Yes	BB	14
	Yanos et al.	No	0,40	0,57	117	Yes	AA	19
7	Štrkalj Ivezi	Yes	0,61	0,41	80	Yes	AA	13
8	Yılmaz y Kav	Yes	0,66	14,63	69	Yes	BB	19
9	Tang et al. (	Yes	0,78	4,60	59	Yes	AA	22

Forest plot Yanos et al. (2019)

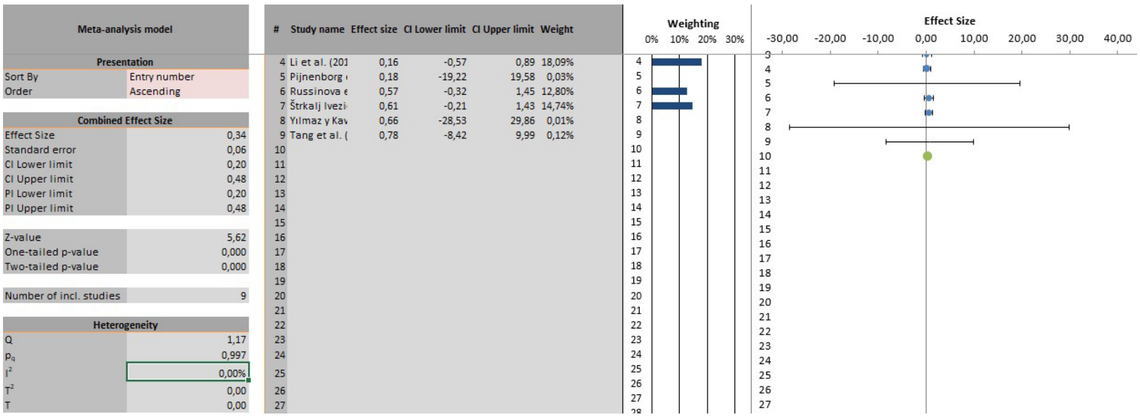

Input Yılmaz y Kavak (2020).

#	Study name	Include study	Effect size	Standard error	Number of observations (for CIs)	Sufficient data	Subgroup	Moderator
1	Frączek-Cen	Yes	0,33	0,30	77	Yes	AA	15
2	Drapalski e	Yes	0,25	0,40	216	Yes	AA	16
3	Huang et al	Yes	0,14	0,47	86	Yes	AA	13
4	Li et al. (201	Yes	0,16	0,37	327	Yes	AA	18
5	Pijnenborg	Yes	0,18	9,80	121	Yes	BB	20
6	Russinova e	Yes	0,57	0,44	46	Yes	BB	14
7	Yanos et al.	Yes	0,40	0,57	117	Yes	AA	19
8	Štrkalj Ivezi	Yes	0,61	0,41	80	Yes	AA	13
	Yılmaz y Ka	No	0,66	14,63	69	Yes	BB	19
9	Tang et al. (	Yes	0,78	4,60	59	Yes	AA	22

Forest plot Yılmaz y Kavak (2020).

Meta-analysis model	
**Presentation**	
Sort By	Entry number
Order	Ascending
**Combined Effect Size**	
Effect Size	0,34
Standard error	0,06
CI Lower limit	0,21
CI Upper limit	0,48
PI Lower limit	0,21
PI Upper limit	0,48
Z-value	5,88
One-tailed p-value	0,000
Two-tailed p-value	0,000
Number of incl. studies	9
**Heterogeneity**	
Q	1,19
p_Q	0,997
I^2	0,00%
T^2	0,00
T	0,00

#	Study name	Effect size	CI Lower limit	CI Upper limit	Weight
4	Li et al. (201	0,16	-0,57	0,89	16,81%
5	Pijnenborg (	0,18	-19,22	19,58	0,02%
6	Russinova e	0,57	-0,32	1,45	11,89%
7	Yanos et al.	0,40	-0,73	1,53	7,08%
8	Štrkalj Ivezi	0,61	-0,21	1,43	13,69%
9	Tang et al. (	0,78	-8,42	9,99	0,11%
10					
11					
12					
13					
14					
15					
16					
17					
18					
19					
20					
21					
22					
23					
24					
25					
26					
27					

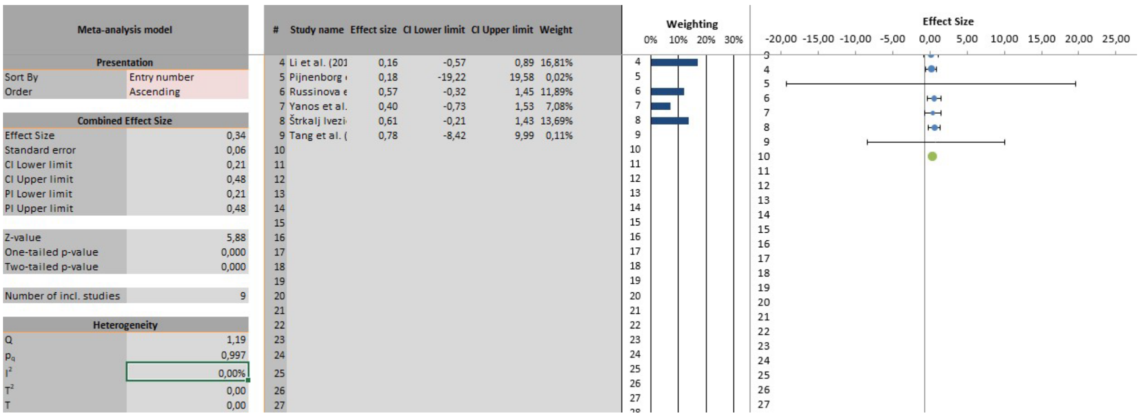

Supplementary File S4-S5. Detailed Characteristics of Included Studies

Supplementary Tables. Characteristics of studies included in the qualitative synthesis

Table S1. Study quality was assessed using the Critical Appraisal Skills Programme España (CASPe). Levels of evidence were classified according to the Scottish Intercollegiate Guidelines Network (SIGN).

First Author & Year	Design	Data Collection	Objectives	Location and Date of Execution	Population and Sample	Results	Conclusions	Quality of the Study
**Ma et al. [59]**	Longitudinal descriptive.	Self-administered sociodemographic questionnaire (age, sex, educational level, marital status, diagnosis, and duration of illness). Scales: Social Support Rating Scale (SSRS), Internalized Stigma of Mental Illness Scale (ISMI), Positive and Negative Syndrome Scale (PANSS), and Personal and Social Performance Scale (PSP).	To examine the prospective associations between social support and three key variables in patients with severe mental illness (SMI) in the community recovery phase: self-stigma, psychiatric symptoms, and personal and social functioning.	China, September 2021 to April 2022.	**Population:** Adults (18–60 years) diagnosed with schizophrenia or bipolar disorder in the community recovery phase in China. **Sample:** 312 participants at baseline assessment.	Higher baseline levels of social support were significantly associated with a reduction in self-stigma ($\beta = -.115$, $p = .029$), a decrease in psychiatric symptoms ($\beta = -.193$, $p < .001$), and an improvement in personal and social functioning ($\beta = .134$, $p = .008$) at 6-month follow-up. In addition, self-stigma partially mediated the relationship between social support and psychiatric symptoms (indirect effect: $\beta = -.043$, 95% CI [−0.074, −0.018]). Furthermore, self-stigma and psychiatric symptoms jointly mediated the relationship between baseline social support and personal and social functioning at 6 months (indirect effect: $\beta = .084$, 95% CI [0.029, 0.143]).	Social support plays a crucial role in improving psychiatric symptoms and personal and social functioning among individuals with severe mental illness (SMI) in the community recovery phase. In addition, self-stigma acts as a key mediating mechanism in these effects, particularly in the relationship between social support and psychiatric symptoms. Community-based interventions aimed at strengthening social support and reducing self-stigma are recommended to improve long-term outcomes in this population.	HIGH
**Villotti et al. [64]**	Longitudinal descriptive.	Self-administered sociodemographic questionnaire. Scales: Job Content Questionnaire (JCQ), Internalized Stigma of Mental Illness Scale (ISMI), Job Tenure Self-Efficacy Scale, and Endicott Work Productivity Scale (EWPS).	To examine a serial mediation model explaining how workplace social support influences work productivity among individuals with severe mental illness, through two mediating variables: workplace self-stigma and job tenure self-efficacy.	Canada, 2012-2016.	**Population:** Individuals aged over 18 years with severe mental illness (e.g., schizophrenia, bipolar disorder) employed in social enterprises. **Sample:** 170 participants at baseline assessment.	Perceived workplace social support was positively associated with higher perceived work productivity, and this relationship was fully mediated by lower levels of workplace self-stigma and higher levels of job tenure self-efficacy. The total indirect effect was significant ($\beta = 5.12$, 95% CI [2.88, 8.44]), and the serial indirect effect through both mediators was also significant ($\beta = 1.01$, 95% CI [0.42, 2.28]). The inclusion of	Workplace social support enhances perceived work productivity among individuals with severe mental disorders by reducing self-stigma and strengthening job tenure self-efficacy. These findings underscore the importance of inclusive work environments and psychological interventions aimed at reducing self-stigma and increasing work-related confidence as key strategies to facilitate employment integration and psychosocial recovery in this population.	MEDIUM

						the mediators rendered the direct effect of social support on productivity non-significant, indicating full mediation.		
**Kim et al. [47]**	Cross-sectional descriptive	Face-to-face structured interviews. Scales: Internalized Stigma of Mental Illness Scale (ISMI), Rosenberg Self-Esteem Scale (RSES), Beck Hopelessness Scale (BHS), Scale of Social Support (SSS), Negative Social Interaction Scale (NSIS), Scale to Assess Unawareness of Mental Disorder (SUMD), Brief Psychiatric Rating Scale (BPRS), Young Mania Rating Scale (YMRS), Montgomery–Åsberg Depression Rating Scale (MADRS), and Global Assessment of Functioning (GAF).	To explore psychosocial factors associated with self-stigma in Korean outpatients with schizophrenia or major affective disorder, with particular attention to their relationships with psychiatric symptoms, self-esteem, social support, insight, and psychosocial functioning.	South Korea. Does not specify date of completion.	**Population:** Outpatients aged 18–65 years diagnosed with schizophrenia or major affective disorder (major depressive disorder or bipolar disorder), receiving care at psychiatric hospitals in South Korea, clinically stable and capable of participating in structured interviews. **Sample:** 229 patients.	High levels of internalized stigma were observed in 8.1% of Korean patients with severe mental illness. Internalized stigma was significantly associated with lower self-esteem, greater hopelessness, higher social conflict, greater insight, a higher number of hospitalizations, and higher educational level. No significant differences in stigma levels were found according to diagnosis (bipolar disorder vs. schizophrenia) or between inpatient and outpatient settings.	Internalized stigma among Korean patients with severe mental illness is primarily influenced by psychosocial variables such as self-esteem, hopelessness, and social conflict, rather than by clinical or demographic factors. Psychosocial interventions that promote self-esteem, hope, and coping skills for managing social conflict, particularly within the family context, may be effective in reducing internalized stigma in this population.	MEDIUM
**Ran et al. [50]**	Cross-sectional descriptive	Face-to-face structured interviews with the patient and a key informant. Sociodemographic and clinical questionnaire (PIFS). Diagnosis according to ICD-10 criteria, conducted by trained psychiatrists. Scales: Internalized Stigma of Mental Illness Scale (ISMI).	To examine the prevalence of self-stigma among individuals with severe mental illness living in rural areas of China and to analyze its association with sociodemographic, clinical, functional, and quality-of-life variables, in order to identify factors significantly related to higher levels of self-stigma in this population.	China**,** entre september to october 2015.	**Population:** Individuals aged 22–91 years with severe mental disorders (primarily schizophrenia and major affective disorder) living in rural areas of Sichuan Province, China, and able to participate in interviews. **Sample:** 453 participants.	Moderate to severe levels of self-stigma were observed in 94.7% of participants. Individuals with schizophrenia showed significantly higher scores on the total ISMI scale, as well as on the alienation and discrimination experience subscales, compared to those with bipolar disorder. Lower family income was associated with higher self-stigma among individuals with schizophrenia and major depressive disorder. Predictors of self-stigma varied across diagnostic groups.	Self-stigma is common and severe among individuals with severe mental disorders in rural Chinese communities, particularly among those with low income. Individuals with schizophrenia tend to experience higher levels of self-stigma than those with bipolar disorder. The development of tailored, individualized interventions is recommended to reduce self-stigma in these populations.	MEDIUM
**Liu et al. [56]**	Cross-sectional observational	Validated and standardized questionnaires in Chinese, self-administered or administered with assistance when required, and completed in clinical settings under the supervision of trained researchers. Scales: Internalized Stigma of Mental Illness	To explore psychosocial factors associated with self-stigma among individuals with severe mental illness in China, specifically examining the relationships between self-stigma and variables such as self-esteem, hope, perceived social support, and the severity of psychiatric symptoms, in order to identify potential predictors	China. Does not specify date of completion.	**Population:** Adults aged 18–69 years receiving psychiatric care in five communities in Huaian, Jiangsu Province, China. **Sample:** 202 participants.	Some degree of self-stigma was observed in 50.5% of participants, with 19.8% exhibiting moderate to severe levels. The highest scores were found in the subscales of value devaluation, discrimination experience, and social withdrawal. Self-stigma was significantly associated with greater perceived discrimination, lower social support, lower family income,	Self-stigma is common among individuals with severe mental illness in China. Psychosocial factors such as perceived discrimination, low social support, negative coping strategies, and economic status significantly influence its intensity. Mental health professionals, particularly psychiatric nurses, are encouraged to develop targeted interventions to reduce self-stigma and promote recovery.	HIGH

		Scale (ISMI), Perceived Devaluation–Discrimination Scale (PDD), Coping With Stigma Scale, and Medical Outcomes Study Social Support Survey (MOS-SS).	that may inform future clinical interventions.			older age, and greater use of distancing coping strategies.		
**Cunningham et al. [53]**	Cross-sectional observational	Combination of self-report measures and administered assessments to evaluate cognitive and psychosocial variables. Scales**:** Self-Stigma of Mental Illness Scale (SSMIS), Maryland Assessment of Recovery in People With Serious Mental Illness (MARS), and Beck Cognitive Insight Scale (BCIS).	To examine how the four components of the social–cognitive model of self-stigma (awareness, agreement, application, and harm to self-esteem) are related to recovery orientation in adults with severe mental illness. Cognitive insight was assessed as a potential moderator of this relationship, in order to identify possible intervention mechanisms to reduce the negative impact of self-stigma on the recovery process.	United States. Does not specify date of completion.	**Population:** Adults with severe mental illness receiving care in psychosocial rehabilitation services at community centers in Maryland. **Sample:** 268 participants.	Self-application of stereotypes and reduced self-esteem were the components of self-stigma most strongly associated with lower recovery orientation. In addition, cognitive insight moderated this relationship, such that the negative impact of self-stigma on recovery was stronger among individuals with lower levels of cognitive insight.	The study confirms that the cognitive and emotional components of self-stigma negatively affect recovery orientation. Interventions that promote cognitive flexibility and reduce stigma-related shame may improve mental health outcomes. Cognitive insight emerges as a relevant protective factor.	HIGH
**Cullen et al. [52]**	Cross-sectional observational	Face-to-face structured interviews conducted by trained interviewers. Scales: Recovery Assessment Scale (RAS), Internalized Stigma of Mental Illness Scale (ISMI), PANSS to assess symptom severity, and the ECA Study Instrument to evaluate the size and quality of the social network.	To examine the relationship between the size and perceived quality of social networks, internalized stigma, and recovery-oriented attitudes in individuals with severe mental illness, in order to inform clinical interventions aimed at strengthening social networks and social support to improve outcomes in this population.	United States. August 2008 to December 2012.	**Population:** Adults with severe mental illness receiving care in community mental health services in the United States. **Sample:** 271 participants.	Participants exhibited small social networks and low levels of perceived social support. A greater number of friends and higher levels of support from family and friends were associated with greater personal confidence, hope, and goal orientation. Additionally, a greater number of friends and family members and higher family support were associated with lower levels of internalized stigma. Frequency of contact was not significantly related to stigma or recovery-oriented attitudes.	The size and perceived quality of social networks are significantly related to internalized stigma and recovery-oriented attitudes among individuals with severe mental illness. These findings suggest that clinical interventions aimed at strengthening social networks and social support may improve outcomes in this population.	MEDIUM
**Hack et al. [55]**	Cross-sectional observational	Individual face-to-face interviews conducted by trained personnel. Scales: Service Engagement Scale (SES), Wahl Stigma and Discrimination Scale (WSD), and Self-Stigma of Mental Illness Scale – Self-Concurrence subscale (SSMIS–SC).	To examine how experiences of stigma, discrimination, and self-stigma are associated with active engagement in mental health treatment among adults with severe mental illness, and to explore whether demographic variables such as age, gender, race, and educational level moderate these relationships. The	United States. Does not specify date of completion.	**Population:** Adults with severe mental illness receiving care in community mental health services in the United States. **Sample:** 167 participants.	Overall, no significant correlations were found between treatment engagement and experiences of stigma, discrimination, or self-stigma. However, educational level moderated these relationships: among individuals with higher education, perceived stigma was associated with greater engagement, whereas self-	Internalized stigma may negatively affect treatment engagement among individuals with higher educational levels, whereas perceived stigma may have a motivating effect within this same group. These findings highlight the need for targeted interventions to prevent or address self-stigma, particularly among individuals with higher educational attainment.	HIGH

		Self-reported demographic data (age, gender, race, and educational level).	study aims to enhance understanding of factors influencing proactive treatment participation beyond access or attendance, in order to inform interventions that promote greater therapeutic engagement.			stigma was associated with lower engagement.		
**Grover et al. [54]**	Cross-sectional observational	Individual face-to-face interviews conducted by trained interviewers. Scales: Internalized Stigma of Mental Illness Scale (ISMIS), Participation Scale (P-Scale), PANSS, Hamilton Depression Rating Scale (HDRS), and Young Mania Rating Scale (YMRS). Diagnosis confirmed using the MINI-PLUS interview and DSM-IV criteria.	To assess the level of self-stigma and its associated factors among patients with severe mental disorders (schizophrenia, bipolar disorder, and recurrent depressive disorder) in clinical remission, using a representative multicenter sample from India. The study also aims to compare levels of stigma across diagnostic groups and to examine the influence of sociodemographic and clinical variables on stigma experiences, in order to inform more effective stigma-reduction interventions.	India. Does not specify date of completion.	**Population:** Adults with a clinical diagnosis of severe mental disorder (schizophrenia, schizoaffective disorder, bipolar disorder, or severe depression) receiving care at psychiatric hospitals in India.<br>**Sample:** 1,403 participants.	Patients with schizophrenia reported significantly higher levels of self-stigma compared to patients with affective disorders. Stigma was more prevalent during the early stages of illness and treatment. Higher levels of stigma were reported by women, single individuals, those who were unemployed, had higher educational levels, belonged to nuclear families, lived in rural areas, and were from middle to upper socioeconomic classes. Stigma was associated with greater restrictions in social participation, particularly among individuals with schizophrenia.	Self-stigma is higher among patients with schizophrenia than among those with affective disorders. Stigma is more intense during the early stages of illness, suggesting that interventions should focus on the initial phases of treatment. In addition, specific sociodemographic factors may help identify patients who are more vulnerable to stigma and guide the development of culturally adapted intervention programs.	MEDIUM
**Ouali et al. [57]**	Cross-sectional observational	Self-administered questionnaire adapted to the Tunisian cultural context. Scales: Self-Stigma of Mental Illness Scale (SSMIS), Devaluation–Discrimination Scale (DDS), and Internalized Stigma of Mental Illness Scale (ISMI). Four additional items were developed based on qualitative interviews with patients, with responses recorded on a 4-point Likert-type scale.	To evaluate stigma related to severe mental illness as perceived and experienced by Tunisian patients diagnosed with schizophrenia or bipolar disorder, taking into account the country's specific cultural context. The study aims to examine how these patients internalize, anticipate, and experience stigma, as well as their attitudes toward diagnostic disclosure, in order to provide evidence for the development of culturally adapted stigma-reduction interventions for this vulnerable population.	Túnez. April 2010 to January 2011	**Population:** Adult patients with severe mental disorders who were clinically stable and had not been hospitalized in the previous three months.<br>**Sample:** 105 patients.	Men reported more experiences of stigmatization in the workplace and expressed less favorable views regarding the social reintegration of individuals with mental illness. Despite widespread apprehension about diagnostic disclosure, most patients had disclosed their diagnosis to close contacts, though fewer had done so with their employers.	There is a significant presence of both experienced and internalized stigma among patients with severe mental illness in Tunisia, manifested through feelings of shame, inferiority, and lack of autonomy. Although fear of diagnostic disclosure persists, particularly due to family and employment concerns, many patients choose to disclose their condition. These findings highlight the urgent need for anti-stigma campaigns and patient-centered, culturally adapted approaches to reduce endorsed stigma and improve social integration in this vulnerable group.	MEDIUM
**Alonso et al. [62]**	Systematic review	Standardized scales were primarily used to assess internalized stigma and related variables. Most frequently used scales: Internalized	To conduct an updated and rigorous systematic review of the characteristics and effectiveness of psychological and psychosocial interventions aimed at reducing	Spain. Does not specify date of completion.	**Population:** Adults with severe mental disorders.<br>**Sample:** The review included 14 studies, with individual sample sizes ranging from 29 to	Fourteen studies published between 2008 and 2018 were reviewed. Nine of these reported a significant reduction in internalized stigma, with small to moderate effect sizes. The most effective	Effective interventions exist to reduce internalized stigma among individuals with severe mental disorders. The most effective approaches are stigma-focused psychoeducational and multicomponent interventions. Cognitive–behavioral and disclosure-based interventions show less	HIGH

		Stigma of Mental Illness Scale (ISMI), Self-Stigma of Mental Illness Scale (SSMIS), Rosenberg Self-Esteem Scale (RSE), Beck Depression Inventory (BDI), and the Empowerment Scale.	internalized stigma among people with severe mental disorders. Controlled studies published between 2008 and 2018 that implemented targeted interventions with pre- and post-treatment measures were selected. The review aimed to identify which types of interventions are most effective, which additional outcomes benefit (e.g., self-esteem, coping, or subjective recovery), and the methodological characteristics of the existing evidence base.		268 participants per study, for a total of 1,275 individuals evaluated across the included investigations.	interventions were stigma-focused psychoeducational interventions and multicomponent approaches combining psychoeducation, cognitive–behavioral therapy, narrative techniques, motivational components, and peer support. Improvements were also observed in subjective recovery, coping, and self-esteem, although results were heterogeneous.	consistent results. The importance of group-based formats and tailoring interventions to individual characteristics and needs is emphasized.	
**Çapar & Kavak [45]**	Cross-sectional descriptive	Standardized self-report instruments and a sociodemographic characteristics form. Scales: Functional Remission of General Schizophrenia Scale (FROGS) and Internalized Stigma of Mental Illness Scale (ISMIS).	To analyze the effect of internalized stigma on functional recovery among individuals with schizophrenia. A sample of 250 patients in remission was assessed using validated scales measuring both stigma levels and functioning across different areas of daily life. The study aimed to determine whether a significant relationship existed between these factors and how sociodemographic variables such as age, gender, educational level, and employment status influenced this relationship.	Turkey. September 2016 to June 2017.	**Population:** Adult patients diagnosed with schizophrenia and registered at a community mental health hospital. **Sample:** 250 patients.	In a study of 250 patients with schizophrenia in remission, high levels of internalized stigma (mean ISMIS = 80.31) and low levels of functional recovery (mean FROGS = 41.02) were observed. A significant negative correlation was found between the two scales, indicating that higher internalized stigma was associated with lower functional recovery. In addition, higher stigma levels were reported among women, individuals with higher educational attainment, and those who were employed, whereas lower functional recovery was observed among women, individuals with lower educational levels, single participants, and those with lower income.	Internalized stigma has a significant negative impact on functional recovery among individuals with schizophrenia. Educational interventions targeting patients, families, professionals, and the community are recommended to reduce stigma and improve functioning. The key role of psychiatric nursing in the early identification of stigma and in promoting functional recovery is also highlighted.	MEDIUM
**Grover et al. [46]**	Cross-sectional descriptive	Structured questionnaire developed based on previously validated scales. Scales: Internalized Stigma of Mental Illness Scale (ISMI), Explanatory Model Interview Catalogue Stigma Scale (EMIC), Participation Scale (P-Scale), Hamilton Depression Rating Scale (HDRS), Young Mania Rating Scale (YMRS),	To evaluate levels of internalized and perceived stigma and their impact on social participation among patients with bipolar disorder type I in remission, and to analyze their associations with sociodemographic and clinical variables. The study also aims to explore the interrelationships between different forms of stigma and factors such as age, income level, duration of remission, residual depressive	India. Does not specify date of completion.	**Population:** Individuals diagnosed with bipolar disorder type I attending outpatient clinics at a mental health hospital in India. **Sample:** 185 patients.	In a sample of 185 patients with bipolar disorder type I in remission, 29.7% exhibited significant internalized stigma (ISMIS ≥ 2.5), with discrimination experience being the most prevalent dimension (38.9%). Severe restrictions in social participation were reported by 42% of participants. Internalized stigma was associated with younger age, lower income level, shorter duration of remission, a greater	Internalized stigma is highly prevalent among patients with bipolar disorder in India and is associated with clinical factors such as residual depressive symptoms, lower functioning, and shorter duration of remission. There is a clear need for interventions aimed at reducing stigma, particularly within sociocultural contexts in which the family plays a central role in treatment. Addressing stigma may enhance social participation and community integration among patients.	MEDIUM

		and Global Assessment of Functioning (GAF).	symptoms, and overall functioning within the specific cultural context of India.			number and longer duration of depressive episodes, higher residual depressive symptoms, and lower levels of functioning. A positive correlation was also found between internalized stigma, perceived stigma, and restrictions in social participation.		
**Morgades-Bamba et al. [49]**	Cross-sectional descriptive	Standardized questionnaires administered before and after the intervention in both the experimental and control groups. Scales: Internalized Stigma of Mental Illness Scale (ISMI), Rosenberg Self-Esteem Scale (RSES), General Self-Efficacy Scale (GSES), and Positive and Negative Affect Schedule (PANAS).	To analyze how internalized stigma affects affective well-being among individuals with schizophrenia, considering the mediating roles of self-esteem and self-efficacy. Specifically, the study explored the impact of two key dimensions of internalized stigma—alienation and stereotype endorsement—on positive and negative affect. The proposed model posits that these forms of stigma impair self-concept, which in turn negatively influences emotional well-being. The study aimed to provide empirical evidence for this process to inform clinical interventions that promote recovery and help prevent adverse outcomes such as suicide.	Spain. Does not specify date of completion	**Population:** Individuals diagnosed with schizophrenia according to DSM-5 criteria. **Sample:** The study included 216 participants.	In a sample of 216 individuals with schizophrenia, both alienation and stereotype endorsement—dimensions of internalized stigma—were negatively associated with self-esteem and self-efficacy, which in turn influenced affective well-being. Self-esteem mediated the effects of both stigma dimensions on positive and negative affect, whereas self-efficacy mediated only the effect of alienation on positive affect. Alienation showed a stronger impact than stereotype endorsement.	Internalized stigma, particularly alienation, undermines affective well-being among individuals with schizophrenia through reductions in self-esteem and self-efficacy. Clinical interventions should focus on preventing alienation and strengthening a positive self-concept from the time of diagnosis, in order to promote recovery and reduce suicide risk. The findings also suggest avoiding overly biologicist approaches that reinforce perceptions of schizophrenia as a chronic and incurable condition.	HIGH
**Violeau et al. [51]**	Cross-sectional descriptive	Self-report instruments and structured clinical interviews, administered in a single session to each participant. Scales: Internalized Stigma of Mental Illness Scale (ISMI), Subjective Scale to Investigate Cognition in Schizophrenia (SSTICS), and Rosenberg Self-Esteem Scale (RSE).	To analyze how internalized stigma affects affective well-being among individuals with schizophrenia, considering the mediating roles of self-esteem and self-efficacy. Using a path analysis model, the study aimed to examine how internalized stigma undermines self-concept and, consequently, reduces positive affect and increases negative affect, which are key factors in recovery and suicide prevention in this population.	France. Does not specify date of completion.	**Population:** Individuals diagnosed with schizophrenia or schizophrenia spectrum disorders receiving outpatient follow-up in mental health services in France. **Sample:** The study included 78 participants.	In a sample of 78 individuals with schizophrenia or schizoaffective disorder, metacognitive complaints (subjective perceptions of cognitive difficulties) were found to fully mediate the relationship between internalized stigma and self-esteem. That is, stigma did not directly affect self-esteem but exerted its effect through perceived cognitive inefficacy. Mediation analysis showed a significant indirect effect (ACME = −0.091, $p < .001$), with no significant direct effect of stigma on self-esteem.	Internalized stigma reduces self-esteem among individuals with schizophrenia by negatively influencing their subjective perception of cognitive functioning (offline metacognition). These findings suggest that stigma-reduction interventions should include components addressing subjective cognitive complaints, as they may represent a key mechanism underlying reduced self-esteem. Metacognition should therefore be considered a relevant therapeutic target in recovery-oriented programs.	MEDIUM
			To examine the relationship between peer contact, social support, and self-stigma	China. Does not specify	**Population:** Individuals with severe mental disorders.	Moderate to severe levels of internalized stigma were observed in 81.1% of	Positive peer contact can reduce internalized stigma among individuals with severe mental illness, with social support playing a key mediating role in this relationship.	MEDIUM

**Li et al. [48]**	Cross-sectional descriptive	Scales assessing perceptions of peer contact and media exposure, evaluated using Likert-type items. Scales: Internalized Stigma of Mental Illness Scale (ISMI), Specific Level of Functioning Scale (SLOF), and Duke Social Support Index (DSSI).	among individuals with severe mental illness (SMI) in Hong Kong. Specifically, the study aimed to assess levels and characteristics of self-stigma in this population, identify influencing factors, and analyze the mediating role of social support in the relationship between perceived peer contact and self-stigma, within a cultural context in which traditional Chinese values may intensify experiences of internalized stigma.	date of completion.	**Sample:** 159 participants.	participants (n = 159). Internalized stigma was significantly associated with diagnostic category (higher in mood disorders), duration of illness, poorer social functioning, and negative perceptions of peer contact. Mediation analysis indicated that social support significantly mediated the relationship between peer contact and internalized stigma. Perceptions of media exposure were not associated with stigma.	Peer-based contact interventions are recommended as an effective strategy to reduce stigma, particularly in cultural contexts such as Hong Kong, where traditional values may intensify self-stigmatization.

Supplementary Table. Characteristics of studies included in the meta-analysis

Table S2. Study quality was assessed using the Critical Appraisal Skills Programme España (CASPe). Levels of evidence were classified according to the Scottish Intercollegiate Guidelines Network (SIGN).

First Author & Year	Design	Data Collection	Objectives	Location and Date of Execution	Population and Sample	Results	Conclusions	Quality of the Study
**Frączek-Cendrowska et al. [43]**	Randomized experimental	A battery of standardized instruments was used to collect data before (T0) and after (T1) the intervention, all of which had validated Polish versions. Scales: Internalized Stigma of Mental Illness Scale (ISMI), Stigma Resistance Scale (SRS), Secrecy Scale (SS), Rosenberg Self-Esteem Scale (RSES), Sense of Coherence Scale (SOC-29), De Jong Gierveld Loneliness Scale (DJGLS), Satisfaction With Life Scale (SWLS), and Brief Psychiatric Rating Scale (BPRS).	To evaluate the effectiveness of a group-based cognitive–behavioral therapy (CBT) intervention, entitled *"I Am What I Am"*, aimed at reducing self-stigma and enhancing personal recovery-related resources among hospitalized individuals with severe mental disorders. Specifically, the study sought to determine whether this intervention improved self-esteem, stigma resistance, and sense of coherence compared with treatment as usual in a psychiatric hospital setting.	Poland, May 2021 to July 2022.	Population: Individuals aged 18 years or older with severe mental disorders who were hospitalized but clinically stable and able to provide informed consent. **Sample:** 104 participants.	The experimental group showed significant improvements in stigma resistance (SRS) and sense of coherence (SOC-29) compared with the control group. Both groups exhibited significant reductions in internalized stigma (ISMI), although no significant between-group differences were observed for this variable. No significant differences were found in loneliness, life satisfaction, psychiatric symptoms, or secrecy. The intervention was well tolerated, with a dropout rate of 26%.	The group-based cognitive–behavioral therapy intervention "I Am What I Am" was effective in enhancing key personal resources such as stigma resistance and sense of coherence, which are fundamental to clinical and personal recovery. Although no significant differences were observed in reductions of internalized stigma compared with the control group, the findings suggest that complementing intervention protocols with techniques aimed at strengthening personal resources may be beneficial in hospital settings.	HIGH
**Tang et al. [41]**	Randomized experimental	Structured self-report questionnaires administered before and after the intervention, using validated scales. Scales: Link's Stigma Scales, Five Facet Mindfulness Questionnaire (FFMQ), and Insight and Treatment Attitudes Questionnaire (ITAQ).	To evaluate the effects of mindfulness-based cognitive therapy (MBCT) on stigma among women with schizophrenia, specifically examining how this intervention influences stigma perception, coping strategies, levels of mindfulness, and attitudes toward treatment, in order to determine its effectiveness as a complementary therapeutic tool.	China. Does not specify date of completion.	**Population:** Adult women diagnosed with schizophrenia in a stable phase and hospitalized. **Sample:** 62 patients.	Following the mindfulness-based cognitive therapy (MBCT) intervention, the experimental group showed a significant reduction in perceived stigma and negative coping strategies compared with the control group. In addition, significant increases were observed in levels of mindfulness (FFMQ) and in insight and attitudes toward treatment (ITAQ). Negative correlations were identified between mindfulness and stigma, and positive correlations between mindfulness and treatment attitudes, suggesting that higher levels of mindfulness are associated with lower stigma	Mindfulness-based cognitive therapy (MBCT) is effective in reducing stigma among women with schizophrenia, improving coping orientation, increasing illness awareness, and fostering a positive attitude toward treatment. Levels of mindfulness appear to act as a protective factor against stigma, and MBCT is a promising intervention that warrants promotion in the treatment of individuals with schizophrenia.	HIGH

						and more favorable therapeutic attitudes.		
**Drapalski et al. [44]**	Randomized experimental	alidated self-report scales administered before and after the intervention. Scales: Internalized Stigma of Mental Illness Scale (ISMI), Self-Stigma of Mental Illness Scale (SSMIS), Maryland Assessment of Recovery in Serious Mental Illness (MARS), Sense of Belonging Instrument (SOBI), General Self-Efficacy Scale (GSES), and Brief Symptom Inventory (BSI).	To evaluate the effectiveness of the group-based "Ending Self-Stigma" (ESS) intervention in reducing internalized stigma among individuals with severe mental illness, compared with an active control intervention focused on general health and well-being. The study aimed to determine whether ESS produced greater improvements in stigma, self-efficacy, recovery, and sense of belonging, and whether these effects were maintained at six months, particularly among subgroups with significant psychotic symptoms..	United States, October 2011 to May 2014.	**Population:** Adults diagnosed with severe mental illness receiving care in community mental health services. **Sample:** 248 participants.	Both groups (the Ending Self-Stigma intervention and the health and wellness control group) showed significant but modest reductions in internalized stigma and improvements in sense of belonging. No significant between-group differences were observed for either primary or secondary outcomes. However, exploratory analyses indicated that participants with higher baseline levels of psychotic symptoms experienced greater reductions in internalized stigma when assigned to the Ending Self-Stigma intervention compared with the control group. Mean attendance was moderate, and facilitator adherence and competence were high in both conditions.	Both stigma-specific interventions and those that address stigma indirectly may be useful in reducing internalized stigma among individuals with severe mental illness. However, individuals with significant psychotic symptoms appear to benefit more from targeted interventions such as Ending Self-Stigma. These findings highlight the need for future research to identify which interventions are most effective, for whom, and under what contextual conditions.	HIGH
**Tang et al. [40]**	Randomized experimental	Standardized instruments administered before and after the intervention. Scales: Perceived Devaluation–Discrimination Scale (PDD), Herth Hope Index Scale (HHIS), Simplified Coping Style Questionnaire (SCSQ), and Quality of Life Scale for Schizophrenia (SQLS).	To evaluate the effect of a positive psychology–based expressive writing intervention (PPEW) on stigma, hope, coping style, and quality of life among hospitalized women with schizophrenia. The study hypothesized that by promoting emotional expression and positive thinking, this intervention could reduce internalized stigma and improve psychological and functional well-being.	China, June 2019 to May 2020.	**Population:** Adult women diagnosed with severe mental illness who were hospitalized. **Sample:** 54 participants.	The experimental group, which received a two-week positive psychology–based expressive writing (PPEW) intervention, showed significant reductions in perceived stigma as well as improvements in hope, positive coping, and quality of life compared with the control group receiving routine psychiatric care. These differences were statistically significant in both per-protocol and intention-to-treat analyses. Effects were particularly pronounced in the domains of positive coping, psychosocial symptoms, and hope.	The study concludes that the positive psychology–based expressive writing (PPEW) intervention is an effective, low-cost, and feasible strategy for reducing stigma, increasing hope, improving coping styles, and enhancing quality of life among hospitalized women with schizophrenia. Its incorporation into routine psychological care is recommended, particularly in clinical settings with limited resources, and further studies with larger samples and long-term follow-up are warranted.	HIGH
		Standardized questionnaires administered by trained researchers. Scales: Internalized Stigma of Mental Illness Scale (ISMIS),	To evaluate the effects of the NECT intervention on perceived stigma, depressive symptoms, self-esteem, and hope among hospitalized individuals with schizophrenia in Taiwan. The intervention combined			The group that received the Narrative Enhancement and Cognitive Therapy (NECT) intervention showed a significant reduction in internalized stigma at the end of the 20-week program, particularly on the "Stopping Self" subscale of the DISC, which assesses self-exclusion	NECT therapy proved effective in reducing perceived stigma among individuals with schizophrenia, particularly in aspects related to self-exclusion. Although no significant improvements were observed in self-esteem, hope, or depression, the findings suggest that personal narrative and cognitive restructuring may strengthen positive identity and prepare patients to cope with stigma in the community following hospital discharge. Its clinical application is recommended as part of the rehabilitation process.	

**Huang et al. [35]**	Randomized experimental	Discrimination and Stigma Scale (DISC-12), Rosenberg Self-Esteem Scale (RSES), Beck Depression Inventory–II (BDI-II), and Herth Hope Index (HHI).	psychoeducation, cognitive restructuring, and narrative techniques, with the aim of reducing internalized stigma and strengthening participants' positive personal identity during preparation for discharge and community reintegration.	Taiwan. Does not specify date of completion.	**Population:** Adults diagnosed with chronic schizophrenia. **Sample:** 86 participants.	behaviors. No significant improvements were observed in self-esteem, hope, or depressive symptoms, possibly due to ceiling effects in baseline scores. The intervention was well tolerated, with a low dropout rate, and positive effects were noted to consolidate during the narrative phase of the program.		HIGH
**Li et al. [36]**	Randomized experimental	Structured interviews including clinical assessment of insight and treatment adherence conducted by psychiatrists. Scales: Internalized Stigma of Mental Illness Scale (ISMI), Discrimination and Stigma Scale (DISC-12), Global Assessment of Functioning (GAF), Schizophrenia Quality of Life Scale (SQLS), Self-Esteem Scale (SES), Brief Psychiatric Rating Scale (BPRS), and PANSS Negative Subscale (PANSS-N).	To evaluate the effectiveness of a comprehensive community-based intervention among individuals with schizophrenia in Guangzhou, China, aimed at reducing anticipated discrimination, improving stigma-coping skills, and promoting improvements in clinical symptoms and social functioning. The intervention combined anti-stigma and anti-discrimination strategies, psychoeducation, social skills training, and cognitive–behavioral therapy, and was compared with standard care over a nine-month period.	China, February to September 2013.	**Population:** Adults diagnosed with schizophrenia residing in urban and rural communities in Guangzhou. **Sample:** 253 participants.	After nine months of intervention, the experimental group showed significant improvements in psychotic symptoms (BPRS, PANSS-N), global functioning (GAF), and stigma-coping skills (DISC subscale). A significant reduction in anticipated discrimination was also observed. However, no significant changes were found in internalized stigma (ISMI), self-esteem (SES), quality of life (SQLS), experienced discrimination, or positive treatment. The intervention combined anti-stigma strategies, psychoeducation, social skills training, and cognitive–behavioral therapy.	A comprehensive community-based intervention can be effective in reducing anticipated discrimination and improving clinical symptoms and social functioning among individuals with schizophrenia. However, no significant effects were observed on internalized stigma or self-esteem, suggesting that these domains may require more specific or intensive interventions. The findings highlight the need to involve family members and the social environment in future strategies to address stigma more comprehensively.	HIGH
**Modelli et al. [37]**	Randomized experimental	Standardized self-report questionnaires. Scales: Coming Out with Mental Illness Scale (COMIS), Cognitive Appraisal of Stigma as a Stressor (CogApp), Authenticity Scale, Self-Stigma of Mental Illness Scale – Short Form (SSMIS), Internalized Stigma of Mental Illness Scale (ISMIS), Rosenberg Self-Esteem Scale (RSES), Subjective Quality of Life (17 items), and Barriers to Access to Care Evaluation (BACE).	To evaluate whether a group-based intervention based on the Honest, Open, Proud (HOP) program could help individuals with mood disorders (depression and bipolar disorder) reduce stigma-related stress, improve self-awareness, particularly self-alienation, and facilitate decision-making regarding diagnostic disclosure, compared with a control group receiving unstructured psychoeducation.	Brazil, between mid-2018 and late 2019.	**Population:** Adults diagnosed with mood disorders (such as depression or bipolar disorder) receiving outpatient mental health care in São Paulo. **Sample:** 61 participants.	The HOP program did not produce significant changes in decisions regarding diagnostic disclosure; however, it showed a marginally significant reduction in stigma-related stress among participants with depression, and an improvement in the self-alienation subscale of the Authenticity Scale in both groups (depression and bipolar disorder). No significant differences were observed on other outcome measures. Participants reported positive evaluations of the group experience and of sharing personal narratives.	The study suggests that the intervention based on the HOP program may be useful in reducing stigma-related stress and improving self-awareness among individuals with mood disorders, particularly those with depressive symptoms. Although it did not alter decisions regarding diagnostic disclosure, the program demonstrated potential as a brief and feasible tool in public health settings to address the emotional impact of stigma.	MEDIUM
**Pijnenborg et al. [38]**	Randomized experimental	Standardized instruments administered in a structured clinical interview format.	To evaluate the effectiveness of the group-based REFLEX intervention in improving clinical insight among	Netherlands, 2012 to 2015.	**Population:** Adults diagnosed with psychosis and impaired clinical	Both groups showed significant improvements in clinical insight following the intervention and at the six-	Structured interventions can improve clinical insight among individuals with schizophrenia, even in chronic phases. Although REFLEX was specifically designed to target factors influencing insight, it did not demonstrate	HIGH

		Scales: Internalized Stigma of Mental Illness Scale (ISMI), Self-Reflection and Insight Scale (S-RIS), Beck Cognitive Insight Scale (BCIS), Theory of Mind subscale of the Davos Assessment of Cognitive Biases Scale (DACOBS), Schedule for Assessment of Insight – Expanded (SAI-E), Item G12 of the Positive and Negative Syndrome Scale (PANSS-G12), Quick Inventory of Depressive Symptomatology – Self-Report (QIDS-SR), Self-Esteem Rating Scale – Short Form (SERS), Manchester Short Assessment of Quality of Life (MANSA), and Positive and Negative Syndrome Scale (PANSS).	individuals with schizophrenia. REFLEX was designed to stimulate three key preconditions of insight: self-reflection, perspective-taking, and sensitivity to stigma. The intervention was compared with an active control condition based on simplified cognitive training, and it was hypothesized that enhancing these capacities would also yield benefits in mood, self-esteem, and quality of life.		insight. **Sample:** 121 participants.	month follow-up. The REFLEX group demonstrated a greater initial improvement in clinician-rated insight; however, this difference was no longer present at follow-up. No significant changes were observed in insight precursors (self-reflection, perspective-taking, or stigma), nor in self-esteem, depression, or psychotic symptoms. Quality of life improved in both groups. The findings suggest that both REFLEX and simplified cognitive training may enhance clinical insight in patients with chronic conditions.	superiority over the active control condition. The findings highlight the need for further investigation into underlying mechanisms and suggest that insight may improve without necessarily modifying its cognitive or social precursors.	
**Russinova et al. [39]**	Randomized experimental	A demographics form was used to collect sociodemographic and clinical characteristics. The *Employment and Vocational Activities Checklist* was used to assess participation in vocational services, and *Belief in One's Own Working Capacity* was included as a single-item measure of work-related self-efficacy. Scales: Work Hope Scale (WHS), Vocational Identity Scale (MVS), Multidimensional Work Motivation Scale (MWMS), Internalized Stigma of Mental Illness Scale (ISMI), and Empowerment Scale.	To evaluate the effectiveness of an innovative group-based intervention called Vocational Empowerment Photovoice (VEP), designed for individuals with psychiatric disabilities who were neither employed nor engaged in vocational services. Grounded in empowerment-based and participatory photography methodologies, the intervention aimed to promote both extrinsic outcomes (such as participation in employment services and employment rates) and intrinsic outcomes (including work-related hope, motivation, self-efficacy, vocational identity, general empowerment, and reduction of internalized stigma). The study sought to determine whether the VEP program could motivate participants to actively consider employment and	United States, February 2012 to January 2014.	**Population:** Adults with severe psychiatric disabilities. **Sample:** 51 participants.	Participation in the Vocational Empowerment Photovoice (VEP) program was associated with a higher rate of engagement in employment services compared with the control group, although this difference diminished at the three-month follow-up. In addition, participants in the VEP group showed significant improvements in general empowerment, self-efficacy, vocational identity, work-related hope, and reductions in internalized stigma. The intensity of program participation was positively correlated with these benefits. No significant differences were observed in employment rates or work motivation.	The VEP program shows potential to empower individuals with psychiatric disabilities who, despite an interest in employment, do not access vocational services due to defeatist beliefs. The intervention proved effective in improving key intrinsic factors for vocational recovery, such as self-efficacy, work-related hope, and vocational identity, as well as in reducing internalized stigma. Further validation in larger studies is recommended, along with exploration of its application as a complement to supported employment programs.	MEDIUM

			overcome defeatist beliefs that hinder vocational integration.					
**Yanos et al. [42]**	Randomized experimental	Standardized instruments and a structured clinical interview oriented toward diagnosis using the SCID-IV. Scales: Internalized Stigma of Mental Illness Scale (ISMI), Rosenberg Self-Esteem Scale (RSES), Beck Hopelessness Scale (BHS), Quality of Life Scale (QLS), Multidimensional Scale of Independent Functioning (MSIF), Positive and Negative Syndrome Scale (PANSS), Coping with Symptoms Checklist (CSC), and Scale to Assess Narrative Development (STAND).	To evaluate the effectiveness of the group-based Narrative Enhancement and Cognitive Therapy (NECT) intervention in reducing self-stigma among individuals diagnosed with schizophrenia spectrum disorders. The study proposed to compare NECT with an active supportive group therapy (SGT), examining not only its impact on self-stigma but also on related variables such as hopelessness, self-esteem, coping, narrative awareness, psychiatric symptoms, and social functioning. The study further explored whether the care setting (outpatient vs. intensive programs) influenced the effectiveness of the intervention.	United States, October 2014 to December 2017.	**Population:** Adults diagnosed with schizophrenia spectrum disorders receiving treatment in outpatient settings or rehabilitation programs. **Sample:** The study included 170 participants.	Participants in the NECT group showed significant improvements in self-stigma, particularly in the social withdrawal subscale, with more pronounced effects in outpatient settings. Improvements were also observed in hopelessness, avoidant coping, and narrative awareness in more intensive treatment settings. No significant effects were found for psychiatric symptoms or objective social functioning. NECT participants attended more sessions and demonstrated greater therapeutic engagement than those in the control group. Effects were more evident at the three-month follow-up and were maintained at six months.	NECT is effective in reducing self-stigma among individuals with schizophrenia spectrum disorders, particularly in domains such as social withdrawal and narrative re-signification of illness. Although no improvements were observed in symptoms or objective social functioning, NECT showed benefits in coping and therapeutic engagement. Its impact may be enhanced when combined with other targeted interventions, such as supported employment programs. Further research is recommended to examine its long-term effects and its integration with complementary treatments.	HIGH
**Štrkalj Ivezić et al. [61]**	Solomon four-group experimental design	Standardized instruments administered in a self-report format using Likert-type scales ranging from 1 to 4. Scales: Internalized Stigma of Mental Illness Scale (ISMI), Boston University Empowerment Scale (BUES), and Perceived Devaluation and Discrimination Scale (PDD).	To evaluate whether a group-based psychoeducational program grounded in recovery and empowerment principles can reduce self-stigma, increase empowerment, and decrease perceived discrimination among individuals diagnosed with schizophrenia. To this end, an intervention group was compared with a waitlist control group using a Solomon four-group experimental design, which allows control for potential pretest effects.	Croatia. Does not specify date of completion.	**Population:** Adults diagnosed with schizophrenia according to DSM-5 criteria. **Sample:** A total of 80 participants were included.	The group that received the group-based psychoeducational intervention showed a significant reduction in self-stigma levels compared with the control group ($F(1,76) = 8.18$; $p < .01$). No significant effects of the intervention were observed on perceived discrimination or empowerment, although a positive trend was identified for the latter. Analyses confirmed that the effect on self-stigma was independent of pretest exposure, reinforcing the validity of the primary outcome.	Group-based psychoeducation grounded in recovery and empowerment principles proved effective in reducing self-stigma among individuals with schizophrenia. Although no significant effects were observed on empowerment or perceived discrimination, the authors emphasize that the group format facilitates identification with empowered peers rather than with stigmatizing stereotypes. The incorporation of this type of intervention into clinical practice is recommended as part of recovery-oriented programs, particularly to counteract the negative effects of stigma on identity and the therapeutic process.	MEDIUM
**Yılmaz and Kavak [60]**	Quasi-experimental pretest–posttest design without a control group	Sociodemographic information form collecting data on age, gender, marital status, educational level, and income. Scales: Internalized Stigma of	To evaluate whether a mindfulness-based psychoeducational program can reduce levels of internalized stigma among patients with schizophrenia. A 12-session group	Turkey, January 2017 to September 2017.	**Population:** Adults diagnosed with schizophrenia receiving care in a mental health setting in Turkey. **Sample:** The study	The study included 69 patients with schizophrenia (34 in the experimental group and 35 in the control group). Both groups were homogeneous in their sociodemographic characteristics. After 12	Mindfulness-based psychoeducation is effective in reducing internalized stigma among individuals with schizophrenia. This group intervention, which combines illness-related education, mindfulness techniques, and psychodynamic elements, promotes acceptance, self-reflection, and positive coping. Integrating this type of	MEDIUM

		Mental Illness Scale (ISMI).	intervention combining mindfulness techniques, illness-related education, and psychotherapeutic activities was implemented. Pre- and post-intervention outcomes were compared between an experimental group and a control group to determine the impact of the intervention on the different dimensions of stigma.		included 26 participants.	sessions of mindfulness-based psychoeducation, the experimental group showed a significant reduction in overall internalized stigma and in the subscales of alienation, stereotype endorsement, discrimination experience, and social withdrawal compared with the control group. No significant changes were observed in the stigma resistance subscale.	program into rehabilitation processes is recommended to enhance recovery and reduce the impact of stigma.	
